# Impact of virulence genes and pathotypes of intestinal pathogenic *Escherichia coli* on gastrointestinal lesions in pre- and post-weaning piglets

**DOI:** 10.3389/fcimb.2025.1704407

**Published:** 2026-01-26

**Authors:** Tomislav Sukalić, Ana Končurat, Sanja Duvnjak, Doroteja Huber, Ana Beck, Miroslav Benić, Boris Habrun, Gordan Kompes, Andrea Humski

**Affiliations:** 1 Animal Disease Diagnostic Laboratory, Croatian Veterinary Institute, Regional Department Križevci, Križevci, Croatia; 2 Laboratory for Bacterial Zoonoses and Molecular Diagnostics of Bacterial Diseases, Department for Bacteriology and Parasitology, Croatian Veterinary Institute, Zagreb, Croatia; 3 Department of Veterinary Pathology, University of Zagreb – Faculty of Veterinary Medicine, Zagreb, Croatia; 4 O-ZNA, Zagreb, Croatia; 5 Laboratory for Mastitis and Raw Milk Quality, Department for Bacteriology and Parasitology, Croatian Veterinary Institute, Zagreb, Croatia; 6 Substrate and Media Preparation Laboratory, Department for Bacteriology and Parasitology, Croatian Veterinary Institute, Zagreb, Croatia; 7 Laboratory for General Bacteriology, Department for Bacteriology and Parasitology, Croatian Veterinary Institute, Zagreb, Croatia; 8 Laboratory for Food Microbiology, Department for Veterinary Public Health, Croatian Veterinary Institute, Zagreb, Croatia

**Keywords:** *Escherichia coli*, pathological lesions, pathotypes, piglets, virulence genes

## Abstract

**Background:**

Pathogenic strains of *Escherichia coli* (*E. coli*) cause colibacillosis in pre- and post-weaning piglets. Fimbrial and non-fimbrial adhesins, as well as heat-labile and heat-stable enterotoxins, are main virulence factors in enterotoxigenic (ETEC), enteroaggregative (EAEC), enteropathogenic (EPEC) and shigatoxigenic (STEC) pathotypes which cause colidiarrhea or colitoxemia in piglets.

**Methods:**

Fifty-five piglets submitted for necropsy were examined for gross and histological lesions. *E. coli* strains were isolated, biochemically confirmed, and tested by PCR for 15 virulence genes (VGs). Statistical analyses used appropriate parametric or non-parametric tests, depending on the distribution. The results with p values less than or equal to 0.05 (p ≤ 0.05) were considered statistically significant.

**Results:**

Overall, 84.48% of strains carried at least one VG. The occurrence of six VGs - *astA*, *estII*, *faeG*, *estI*, *elt*, and *paa* - was high, with frequencies of 67.24%, 63.97%, 55.18%, 50.00%, 48.27%, and 44.82%, respectively. ETEC predominated (63.79%), while 5.17% of strains carried EPEC or STEC genes; 15.52% were non-specific virotypes, and 15.52% were apathogenic. Lesions were most prominent in the small intestine. The virotype LT:STa:STb:EAST1:PAA:F4 was most common, whereas STa:Stx2:Stx2e was linked to the most severe lesions. Lesions varied depending on the pathotype involved and the VGs expressed. Severity of lesions differed significantly between suckling and weaned piglets (p = 0.0091) and between piglets with and without diarrhea (p = 0.0223), with suckling and diarrheic piglets showing more pronounced pathological changes. More extensive lesions in ETEC were associated with the acquired *astA* and *paa* genes. Pathoscores were significantly associated with *faeG*/F4 (p = 0.0001), *eltA*/LT (p = 0.0001), *estII*/STb (p = 0.0001), *paa*/PAA (p = 0.0002), and *astA*/EAST1 (p = 0.0029).

**Discussion and conclusions:**

Strong associations between specific VGs - particularly *faeG*, *eltA*, *estII*, *paa*, and *astA* - and higher lesion scores show that VG detection can help predict disease severity and guide interventions. Age-specific interpretation is crucial, as isolates from pre-weaned piglets often carried more VGs and were associated with more severe lesions. This study underscores the value of integrating bacteriological, molecular and histopathological data for accurate diagnosis, especially given the high prevalence of VG-positive and recombinant ETEC strains.

## 1 Introduction

The proliferation of pathogenic strains of *Escherichia coli* (*E. coli*) in the small intestine of pigs leads to colibacillosis. The disease occurs most frequently in the perinatal period and after weaning, and a distinction is made between systemic and intestinal colibacillosis (Robins-Browne et al., 2016; Pokharel et al., 2023). Intestinal colibacillosis, which manifests clinically as colidiarrhea and colitoxemia, is caused by pathotypes collectively known as diarrheagenic *E.coli* (DEC) (Gomes et al., 2016) and they are the leading cause of infectious diarrhea in humans and animals (Guo et al., 2020), while in pigs they cause significant economic losses (Dubreuil, 2021; Tamayo-Legorreta et al., 2021). Pathogenic *E. coli* strains cause approximately 50% of gastroenteropathies occurring before weaning (Cvetnić, 2002), and in the pig population at 4–12 weeks of age, 20% of deaths are due to oedema disease (Fairbrother and Gyles, 2006).

The different forms of colibacillosis are the result of infection with varying pathotypes of *E. coli*, whose pathogenicity depends on the virulence factors (VF) expressed (Naidoo and Zishiri, 2025). The most essential VFs in *E. coli* are fimbrial and non-fimbrial adhesins, thermostable and thermolabile enterotoxins as well as hemolysins, invasins, proteases and endotoxins, which are the “weapons” used to attack the host. Virulence genes (VGs) in *E. coli* can be acquired through horizontal gene transfer, contributing to the rapid evolution and adaptation of *E. coli* strains (Desvaux et al., 2020), as well as the emergence of an increasing number of hybrid pathotypes (Robins-Browne et al., 2016; Paiva et al., 2025), which should be considered emerging pathogens (Nammuang et al., 2024). The gross and microscopic lesions in tissues and organs also depend on the involved pathotype. Research over the last 20 years has shown that 50-80% of *E. coli* strains carry genes for at least one of the VFs (Vu-Khac et al., 2007; De Lorenzo et al., 2018; Tusiime et al., 2024).

Depending on the presence of VGs, the following pathotypes are essential in intestinal colibacillosis in pigs: 1. enterotoxigenic (ETEC) 2. enteroaggregative (EAEC) 3. enteropathogenic (EPEC) 4. shiga-toxigenic (STEC).

ETEC has the ability to colonize the intestinal mucosa and simultaneously produce thermolabile (LT) and/or thermostable (STa, STb, EAST1) enterotoxins, which is a prerequisite for the development of the disease (Brown et al., 2007; Dubreuil, 2008; Dubreuil et al., 2016; Dubreuil, 2021; Kim et al., 2022). ETEC binds to glycoprotein receptors of the mucosa in the small intestine by means of fimbrial adhesins. Fimbriae F4, F5, F6 and F41 are responsible for mediating adhesion in neonates, while F18 (together with F4) is common in post-weaning colibacillosis (Castro et al., 2022; Lin et al., 2022). The adherent bacteria are particularly noticeable on the intestinal villi that cover the Peyer’s patches (Nagy et al., 1992) and they disrupt the water and electrolyte circulation through their effect on the mucosa, which leads to diarrhea and consequent dehydration. ETEC is the most important cause of neonatal diarrhea and diarrhea in weaned pigs (Nagy and Fekete, 2005; Brown et al., 2007; Barros et al., 2023; Tsekouras et al., 2023). In neonatal diarrhea, a yellowish-gray watery content is often found macroscopically in the small intestine (Luppi, 2017), sometimes with an admixture of mucus and blood. Microscopically, the lesions are predominantly seen in the ileum (Sha et al., 2024), where small bacterial clusters or a continuous layer of bacteria is found on the surface of enterocytes. Neutrophils and macrophages are found in the lamina propria of the intestinal villi, and a significantly higher number of bacteria is found on the mucosa of the ileum than in the duodenum and jejunum (Do et al., 2006). Villous atrophy accompanied by crypt hyperplasia is the predominant lesion observed (Sha et al., 2024).

EAEC is defined as a pathotype that produces neither LT nor ST, does not invade epithelial cells, but adheres to the intestinal mucosa with an aggregative adherence pattern (Veilleux and Dubreuil, 2006; Jacobson, 2022). Numerous EAEC VFs are associated with the presence of *aggR* gene, which encodes AggR transcriptional regulator found on the EAEC virulence plasmid pAA (Morin et al., 2013). AggR is the main regulator of EAEC virulence factors (Nataro, 2005), and it is considered the defining criterion for EAEC as a pathotype. *aggR*^+^ strains are commonly referred to as typical EAEC, while *aggR*^-^/*aaiC*^+^ strains are classified as atypical EAEC (Guerrieri et al., 2019). Freire et al. (2022) detected 83.60% *aggR*^+^ strains among *E. coli* strains charcterized as EAEC. Recently, the *afpR* gene, which encodes the regulator of the Afp (aggregate-forming pilus) operon, has been suggested as a molecular marker of certain EAEC strains (Del Carpio et al., 2025). The initial attachment of EAEC to epithelial cells is mediated by aggregative adherence fimbriae (AAFs/I–V), encoded by *aggA*, *aafA*, *agg3A*, *agg4A*, and *agg5A*, all located on pAA (Boisen et al., 2020; Pakbin et al., 2021). Some EAEC strains may produce cytotoxins, including the plasmid-encoded toxin and enterotoxins such as EAST1 (Harrington et al., 2006), but the *astA* gene for EAST1 can also be found in other *E. coli* pathotypes (Zajacova et al., 2012). EAEC increases mucus secretion and mucoid biofilm formation, which are associated with strong intestinal colonization and persistence (de Souza et al., 2025). Although EAEC has rarely been isolated from pigs and is considered primarily a human pathogen, it can induce diarrhea in pigs. Lesions in pigs include villus shortening, hemorrhagic necrosis of the villus tips, and inflammatory changes characterized by edema and mononuclear infiltration of the submucosa (Weintraub, 2007), as well as microvilli vesiculation, enlarged crypt openings, and increased epithelial cell extrusion (Jacobson, 2022). Vacuolization in goblet cells is also evident, suggesting stimulation of mucus secretion (Nataro et al., 1998), and bacterial accumulations are observed in the intestinal lumen together with mucus and cellular debris (Andrade et al., 2010).

EPEC does not produce exotoxins, but has an exceptional ability to adhere to epithelial cells of the terminal region of the small intestine and colon (Naglić et al., 2005). Some strains cause direct damage to the mucosa through the characteristic mechanism of attaching and effacing (AEEC). Macroscopically, a congested colon, reddening of the mucosa and muco-necrotic debris can be seen. There is fibrinous hemorrhagic enteritis in the ileum, and the mesenteric lymph nodes are often enlarged. Microscopically, the enterocytes to which the bacteria adhere are rounded and detach individually or in clusters, revealing villous atrophy in the small intestine or microerosions in the colon. A fusion of the villi in the small intestine and a depletion of the goblet cells in the small and large intestine are possible. There is moderate congestion of the mucosa and local infiltration by neutrophils (Brown et al., 2007) as well as vacuolization of the epithelial cells of the intestinal villi, atrophy, neutrophil infiltration and lymphoid necrosis in the Peyer’s patches (Canal et al., 1999). Destroyed brush borders and firmly adhered bacteria can also be found (Janke et al., 1989).

STEC strains produce Shiga toxins (Stx) and cause hemorrhagic diarrhea in addition to destroying microvilli. They are therefore also known as enterohemorrhagic *E. coli* (EHEC). Strains carrying Shiga toxin genes demonstrate higher cytotoxicity than non-Stx strains (Zhu et al., 2025), they also frequently have *astA* gene for EAST1 (Zweifel et al., 2006), but may lack *eae* gene for intimin and do not always exhibit EPEC features. STEC affect the intestinal and vascular epithelium and causes a specific syndrome in pigs that occurs in weaned piglets – edema disease, in which F18ab fimbriae mediate colonization (Debroy et al., 2009; Baek et al., 2023). Macroscopic changes are mainly found in the spiral colon, sometimes also in the cecum, where fibrinous or fibrinous-hemorrhagic enteritis/typhlitis can occur, as well as in the ileum (Tsukahara et al., 2005). Microscopically, rounded, atrophic, or fused villi can be found, and the brush border is unrecognizable and covered by a layer of gram-negative coccobacilli. In the colon, the lesions extend to the glands, which are dilated and filled with dead epithelium and with leukocytes. The crypts in the small intestine may be elongated, and in severely affected intestines, congestion of the mucosa and submucosa, edema, and microvascular thrombi can be observed (Brown et al., 2007).

Previous studies have identified various *E. coli* pathotypes involved in the development of colibacillosis in piglets, causing digestive system lesions of varying severity. However, they do not describe the specific relationship between *E. coli* VGs and lesions in individual organs and tissues in intestinal colibacillosis. Within the scope of this study, we aim to:

1. provide a detailed pathological and histopathological description of intestinal colibacillosis in pre-weaning and post-weaning piglets;
2. determine the presence of VGs encoding specific virulence factors, including fimbrial and non-fimbrial adhesins, as well as heat-labile and heat-stable toxins;
3. identify the most common *E. coli* pathotypes/virotypes in piglets based on the detected VGs;
4. correlate pathological lesions in piglet colibacillosis with VGs and determine which *E. coli* virotypes are the most pathogenic for piglets and
5. give practical recommendations which can improve the diagnosis and management of colibacillosis in piglets.

## 2 Materials and methods

### 2.1 Material - farms and piglets

Six farms with a total of 365 sows located in Koprivnica-Križevci County, northern Croatia, were selected for sampling. At the time of sampling, neither the sows nor the piglets on these farms were actively immunized with vaccines against *E. coli* infections. Only naturally deceased piglets with suspected colibacillosis, both pre- and post-weaning, up to 12 weeks of age, were collected for the study. Before further analysis, each piglet was examined to determine its suitability for testing. Only fresh carcasses were included; piglets showing signs of autolysis or putrefaction were excluded, as well as crushed piglets. Of the 91 carcasses collected, 55 were deemed suitable and processed for further laboratory analysis.

### 2.2 Methods

#### 2.2.1 Necropsy and sampling for further laboratory tests

The necropsy protocol included an external examination of each carcass to determine age, condition, and sex. Each piglet was assigned a unique identification code (P1–P55) according to the order of necropsy. A necropsy was performed on all animals, focusing on the examination of target organs and tissues such as the stomach, jejunum, ileum, colon, mesentery and mesenteric lymph nodes. All pathological findings were recorded in written form and as digital photographs.

During necropsy, samples from the target organs and tissues were collected for bacteriological and histological examination. For histological analysis, representative sections (0.5–3 cm thick) of morphologically altered organs and tissues were excised and fixed in 10% neutral buffered formalin. To ensure high-quality histological sections of the tubular organs of the digestive tract, the jejunum, ileum, and colon were not opened longitudinally. Instead, 1–3 cm long segments were ligated and gently injected with 10% neutral buffered formalin into the lumen to preserve the intestinal villi. For bacteriological analysis, the contents of the small and large intestines were sampled using sterile equipment to facilitate the isolation of *E. coli*.

#### 2.2.2 Bacteriology

Organs and tissues of the piglets were bacteriologically processed immediately after necropsy. Conventional bacteriological methods were used, and blood agar (Merck, Germany) with an addition of 5% defibrinated sheep blood was used as a base medium for the isolation of *E. coli* to determine the morphological characteristics and hemolytic properties of the individual bacterial strains. Samples were also inoculated onto selective solid nutrient media, MacConkey agar, XLD agar and ENDO agar (Merck, Germany), to differentiate *E. coli* strains from other enterobacteria. The inoculated culture media were incubated at 37 °C/24 hours. Intestinal content was inoculated according to the dilution method to obtain characteristic single bacterial colonies after incubation. The jejunum, ileum and colon of each piglet were inoculated. At the end of incubation, the isolated bacterial cultures were identified based on their characteristic morphology and growth. The VITEK-2 system (Biomerieux, France) with GN cards were used to detect the biochemical properties. The isolated *E. coli* strains were stored on BSS Stock culture agar (Bio-Rad, France) and maintained at 2-8 °C until DNA extraction.

#### 2.2.3 DNA extraction and detection of *Escherichia coli* virulence genes

The bacterial cultures were mixed in 100 μL of sterile distilled water without DNase and RNase (UltraPure™ DNase/RNase-Free Distilled Water, Invitrogen, USA), boiled at 95 °C for 20 minutes and centrifuged at 14,000 g for 1 minute. The supernatant was used as DNA template for the polymerase chain reaction (PCR). PCRs were carried out in 20-μL reactions consisting of 10 μL HotStarTaq Master Mix (Qiagen, Germany), six μL of water (RNase-free water, Qiagen, Germany), 0.5 μM of each primer (Macrogen, The Netherlands), and two μL of template DNA. PCRs were carried out in a Veriti 96-well thermal cycler (Applied Biosystems, USA). The cycling regimes were according to the references, with the initial activation step at 95°C for 15 minutes. The sizes of DNA fragments were determined by QIAxcel capillary electrophoresis (Qiagen, Germany). Results were interpreted according to the references.

All samples (58) were analyzed for the presence of genes responsible for the production of thermolabile and thermostable enterotoxins, Shiga toxins and fimbrial and non-fimbrial adhesins. The genes and virulence factors investigated are listed in **Table 1**, and **Table 2** contains the primers used for the PCR reaction.

**Table 1:** Genes encoding *Escherichia coli* virulence factors.

ADHESINS	FIMBRIAL						NON-FIMBRIAL		
	F4	F5	F6	F18		F41	AIDA- I	PAA	intimin
GENE	*faeG*	*fanC*	*fasA*	*fedA*		*f41*	*aidA*	*paa*	*eae*
TOXINS	THERMOLABILE						THERMOSTABLE		
	LT	Stx1	Stx2		Stx2e		STa	STb	EAST1
GENE	*eltA*	*stx1*	*stx2*		*stx2e*		*estI*	*estII*	*astA*

F4, F5, F6, F18, F41, fimbrial adhesins; LT, termolabile enterotoxin; STa, STb, thermostable enterorotoxins; Stx1, Stx2, Stx2e, Shiga-toxins; EAST1, enteroaggregative thermostable enterotoxin; PAA, porcine attaching and effacing associated protein; AIDA-I, adhesin involved in diffuse adherence.

**Table 2:** The primers used in the study.

Gene	Nucleotide sequences 5>3		Base pair size (bp)	Reference
*faeG*	TGA ATG ACC TGA CCA ATG GTG GAA CC GCG TTT ACT CTT TGA ATC TGT CCG AG		478	Zhang et al. (2007)
*fanC*	CGT ACA TCA ACT ATA GAT CTT GGG CA ATA GAA CCA GAC CAG TCA ATA CGA GCA		137	Zajacova et al. (2012)
*fasA*	GCC AGT CTA TGC CAA GTG GAT ACT TC GTT TGT ATC AGG ATT CCC TGT GGT GG		390	Zhang et al. (2007)
*fedA*	TGG CAC TGT AGG AGA TAC CAT TCA GC ACT TAC AGT GCT ATT CGA CGC CTT AA		268	Zajacova et al. (2012)
*f41*	AGT ATC TGG TTC AGT GAT GGC TGC TG GTA CTA CCT GCA GAA ACA CCA GAT CC		708	Zajacova et al. (2012)
*aidA*	ACA GTA TCA TAT GGA GCC A TGT GCG CCA GAA CTA TTA		586	Li et al. (2020)
*paa*	CCA TAA AGA CAG CTT CAG TGA AAA GTA TTA CTG GTA CCA CCA CCA TCA		162	Li et al. (2020)
*eae*	GAC CCG GCA CAA GCA TAA GC CCA CCT GCA GCA ACA AGA GG		384	Paton and Paton (1998)
*eltA*	TTC CCA CCG GAT CAC CAA CAA CCT TGT GGT GCA TGA TGA		62	EU reference laboratory for *E. coli* EU-RL ETEC Method 008 Rev.0 (EU Reference Laboratory for E. coli, 2013)
*stx1*	ATA AAT CGC CAT TCG TTG ACT AC AGA ACG CCC ACT GAG ATC ATC		180	Paton and Paton (1998)
*stx2*	GGC ACT GTC TGA AAC TGC TCC TCG CCA GTT ATC TGA CAT TCT G		255	Paton and Paton (1998)
*stx2e*	CGG AGT ATC GGG GAG AGG C CTT CCT GAC ACC TTC ACA GTA AAG GT		411	EU reference laboratory for *E. coli* EU-RL VTEC Method 006 Rev.2 (EU Reference Laboratory for E. coli, 2021)
*estI*	STh	GCT AAA CCA GYA GRG TCT TCA AAA CCC GGT ACA RGC AGG ATT ACA ACA	147	EU reference laboratory for *E. coli* EU-RL ETEC Method 008 Rev.0 (EU Reference Laboratory for E. coli, 2013)
	STp	TGA ATC ACT TGA CTC TTC AAA A GGC AGG ATT ACA ACA AAG TT	136	
*estII*	GCT ACA AAT GCC TAT GCA TCT ACA CA CAT GCT CCA GCA GTA CCA TCT CTA AC			Zhang et al. (2007)
*astA*	CCA TCA ACA CAG TAT ATC CGA GGT CGC GAG TGA CGG CTT TGT		111	Yamamoto and Nakazawa (1997)

#### 2.2.4 Histopathology

Representative samples of stomach, jejunum, ileum, colon, mesentery, and mesenteric lymph nodes were fixed in neutral buffered 10% formalin for 24 hours, then routinely processed in Histokinette automatic tissue processor and embedded in paraffin, cut to a thickness of 3 to 5 μm using a sliding microtome and routinely stained with hematoxylin-eosin. The histopathological slides were analyzed using a light microscope. The microscopic morphology of the lesions on histology was recorded using a digital microscopic photographic system, and the recorded digital photographs were processed using the Cell^B software (Olympus, Japan). In the first phase of microscopic analysis, all histology findings were recorded in written and digital format. To facilitate the statistical comparison between histological findings and the results of other examinations, histological diagnoses were graded on a standardized 0–4 scale reflecting the degree of organ damage (Gibson-Corley et al., 2013). Each lesion was assessed using this uniform scoring system, ranging from 0 (no injury) to 4 (severe injury). When multiple lesions of varying severity occurred within the same organ, the highest observed score was used for analysis.

#### 2.2.5 Statistical analysis

Statistical data processing was carried out using the Stata 18 software (Stat Corp., USA). Depending on the data distribution, the results are presented as median, minimum and maximum values or as the arithmetic mean with associated standard deviation. The values were compared using the t-test or the Mann-Whitney test for non-parametric values.

To compare the values of more than two groups, the Kruskall-Wallis test or the analysis of variance was used, depending on the data distribution.

For the comparison of variables expressed in binary form (yes/no), the chi-square test or Fisher’s exact test was used. Correlations between variables were expressed as Spearman rank or Kendall tau b correlation coefficient.

Results with p-values less than or equal to 0.05 (p ≤ 0.05) were considered statistically significant.

#### 2.2.6 Exclusion of co-infections

To exclude hidden co-infections with viral, parasitic and bacterial diseases, a targeted search was carried out for porcine circovirus type 2 (PCV2), *Cystoisospora suis*, *Brachyspira hyodysenteriae*, and anaerobic bacteria of the genus *Clostridium*. These pathogens can cause diarrhea or contribute to starvation and cachexia in piglets of the age groups studied. *Porcine circovirus type 2* (PCV2) was detected using polymerase chain reaction (PCR) with primers specific to the ORF1 region of the viral genome (Platinum^®^ Taq DNA Polymerase, Invitrogen, USA). *Cystoisospora suis*, a common cause of diarrhea in piglets aged 1–3 weeks, and less commonly after weaning, was investigated through examination of small intestine and distal large intestine samples, including their contents. A modified McMaster coprological flotation method was used to identify oocysts. *Brachyspira hyodysenteriae*, the causative agent of swine dysentery, primarily affects older pigs and those in the fattening stage but can, though rarely, be found in weaned piglets. Diagnosis was performed by microscopic examination of fresh native smears from the contents of the large intestine and rectum of weaned piglets.

Enteritis caused by *Clostridioides difficile*, and by *Clostridium perfringens* type C, typically occurs in suckling piglets. For their detection, small intestine samples from these piglets were cultured on blood agar and TSC agar, then incubated under anaerobic conditions using GENbox Anaer (Biomerieux, France) for 48 hours. Finally, specific proliferative and inflammatory changes in the intestinal tract - indicative of transmissible gastroenteritis and proliferative enteropathy (*Lawsonia intracellularis*) - were evaluated histologically.

The farms included in the study were free from brucellosis, classical swine fever, African swine fever, and Aujeszky’s disease. A review of the Croatian Veterinary Institute’s sample database confirmed that no cases of leptospirosis or *Mycoplasma hyopneumoniae* infection had been reported on the selected farms within the past five years.

## 3 Results

### 3.1 Results of bacteriological tests

The small intestines of 55 piglets and the large intestines of 35 piglets were examined bacteriologically. The contents of the jejunum and ileum were inoculated separately. Still, the results of the bacteriological examinations in the small intestine were considered as a whole, as pathogenic strains of *E. coli* can occur in both sections of the small intestine. From the large intestine the contents of the colon were inoculated.

The results are presented in **Table 3**, and it can be seen that *E. coli* strains were isolated from 54/55 (98,18%) bacteriologically positive samples from the small intestine of piglets. Of these, in 31/55 piglets (56.36%) only strains with marked hemolytic properties were isolated, in 19/55 cases only non-hemolytic strains (34.54%), and in four instances both non-hemolytic and hemolytic strains were isolated (7.27%). *Proteus* spp. was isolated in addition to *E. coli* in four cases (7.27%) and a pure culture of ß-hemolytic streptococci was isolated from the small intestine of one piglet.

**Table 3:** Isolated bacterial cultures – intestines.

Culture	*E. coli* Hem	*E. coli* nH	*E. coli* Hem & nH	*Proteus*	Sc ßH	nc
SMI (n=55)	31	19	4	(4)*	1	0
LAI (n=35)	20	8	4	3	0	20

P01-P55, piglets; SMI, small intestines; LAI, large intestines; Hem, hemolytic *Escherichia coli*; nH, non-hemolytic *Escherichia coli*; Sc ßH, beta-hemolytic *Streptococcus* spp.; nc, not cultivated; *Isolated in addition to *E. coli.*

Of the 35 colon samples inoculated, *E. coli* strains with hemolytic properties were isolated in 20/35 cases (57.14%), non-hemolytic *E. coli* in 8/35 cases (22.86%), and both hemolytic and non-hemolytic strains in 4/35 cases (11.43%). In addition to *E. coli* strains, *Proteus* spp. were also isolated from the large intestines of 3/35 (8.57%) pigs.

Considering the entire intestinal tract and the total number of inoculated small and large intestine samples, only hemolytic strains of *E. coli* were isolated from 55/90 (61.11%) of the samples, only non-hemolytic strains from 27/90 (30.00%) of the samples, and both hemolytic and non-hemolytic strains from 8/90 (8.89%). Detailed results are presented in **Supplementary Table 1**.

### 3.2 PCR results for virulence genes

DNA samples from 58 strains of *E. coli* from 54 pigs were analyzed to detect the genes for virulence factors listed in **Table 1**. An exception is piglet P23, from which we did not isolate *E. coli*. Both hemolytic and non-hemolytic strains were isolated from piglets P27, P37, P45 and P47, so two different strains of *E. coli* were processed from each of these piglets. Because previous studies and the present work indicate that lesions in intestinal colibacillosis are predominantly localized in the small intestine, we restricted our analyses to strains isolated from this site. Summarized results of the molecular tests for virulence genes are shown in **Table 4**, while complete results of *E. coli* molecular characterization can be found in **Supplementary Table 2**.

**Table 4:** Summarized results of the molecular tests for *Escherichia coli* virulence genes.

*E. coli* (58 strains from small intestine)	Adhensins								Toxins							Total	Average per strain
	Fimbrial					Non-fimbrial			Termolabile				Termostable				
	F4 *faeG*	F5 fanC	F6 fasA	F18 fedA	F41 f41	AIDA-I aidA	intimin eae	PAA paa	LT eltA	Stx1 stx1	Stx2 stx2	Stx2e stx2e	STa estI	STb estII	EAST1 astA		
VGs detected in 42 *E. coli* strains (suckling piglets)	27	0	7	0	1	2	0	23	25	0	0	0	24	32	32	173	4.11
VGs detected in 16 *E. coli* strains (weaned piglets)	5	0	0	1	0	1	2	3	3	0	1	1	5	5	7	34	2.12
Total VGs detected	32	0	7	1	1	3	2	26	28	0	1	1	29	37	39	207	3.57

F4, F5, F6, F18, F41, fimbrial adhesins; LT, termolabile enterotoxin; STa, STb, thermostable enterorotoxins; Stx1, Stx2, Stx2e, Shiga-toxins; EAST1, enteroaggregative thermostable enterotoxin; PAA, porcine attaching and effacing associated protein; AIDA-I, adhesin involved in diffuse adherence; VG, virulence gene.

Of the 58 strains examined, 49, i.e. 84.48%, carry genes for at least one virulence factor.

Among the fimbrial adhesin genes, the most common was *faeG* (F4), detected in 32/58 cases or 55.17% of the strains, followed by *fasA* (F6) in 7/58 (12.07%) of the strains, *fedA* (F18) and *f41* (F41) in 1/58 (1.72%) of the strains, while the *fanC* F5 gene was not detected in any of the examined strains.

The most frequently found gene in the non-fimbrial adhesins was *paa* (PAA), detected in 26/58 (44.82%) of the strains, while *aidA* (AIDA-I) and *eae* (intimin) are much less common and were found in 3/58 (5.17%) and 2/58 (3.44%) of the strains, respectively.

The *eltA* gene for LT was found in 28/58 (48.27%) of the strains tested, while the genes for Shiga-like toxins – *stx2* for type Stx2 and *stx2e* for subtype Stx2e (specific for edema disease in weaned piglets) – were found in only one strain, accounting for 1.72% of the strains tested. The genes for thermostable toxins are more prevalent, and *astA* (EAST1) and *estII* (STb) are the most common virulence factors in the strains tested, accounting for 39/58 (67.24%) and 37/58 (63.79%) of positive findings, respectively. The *estI* (STa) gene is also frequently present and was found in 29/58 or exactly 50.00% of the strains.

**Figure 1:**
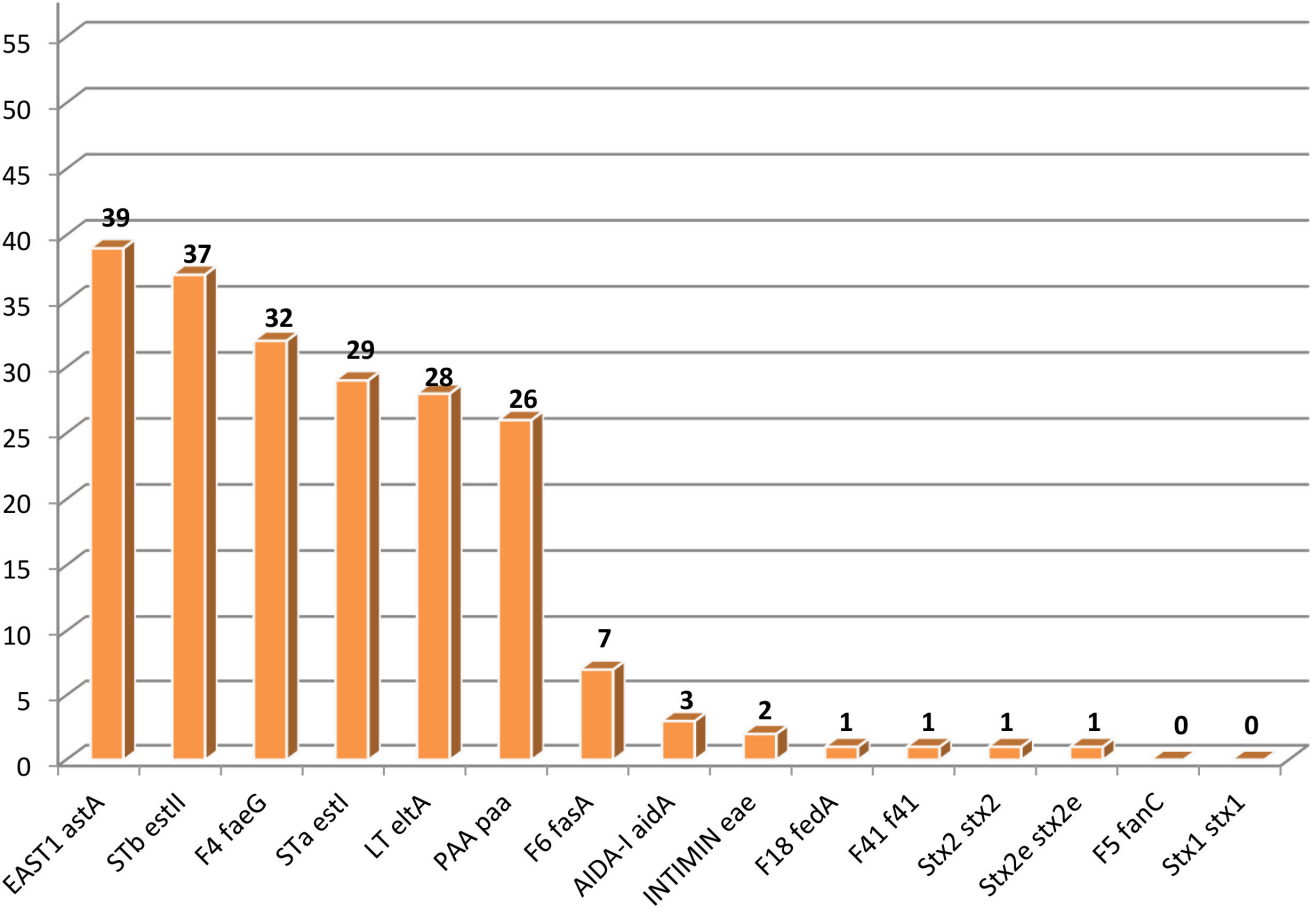
Prevalence of *Escherichia coli* virulence factor genes. F4, F5, F6, F18, F41, fimbrial adhesins; LT, termolabile enterotoxin; STa, STb, thermostable enterorotoxins; Stx1, Stx2, Stx2e, Shiga-toxins; EAST1, enteroaggregative thermostable enterotoxin; PAA, porcine attaching and effacing associated protein; AIDA-I, adhesin involved in diffuse adherence.

The frequency of occurrence of six genes, *astA*, *estII*, *faeG*, *estI*, *eltA*, and *paa* for the virulence factors EAST1, STb, F4, STa, LT and PAA is high, while the other genes tested occur much less frequently or were not detected (**Figure 1**).

#### 3.2.1 Virotypes and pathotypes of *Escherichia coli* identified in the study

The strains of *E. coli* examined in the study yielded 25 different virotypes based on detected VGs, which are presented in **Table 5**. The most common virotype among the 58 strains examined was LT: STa : STb: EAST1:PAA:F4, which occurred 19 times or in 32.76% of cases. The most common pathotype among the strains examined was ETEC (63.79%); 5.17% of strains carried genes associated with the EPEC and STEC pathotypes, while 15.52% of strains were non-specific virotypes. *E.coli* strains which lacked genes for any virulence factor and were considered apathogenic were represented with 15.52%. We found only one hybrid ETEC/STEC strain, virotype STa: Stx2:Stx2e.

**Table 5:** Identified virotypes of *Escherichia coli* in the study.

Number of genes detected	Virotype	Frequency (n=58)
7	LT: STa : STb: EAST1:PAA:F4:F6	1
6	STa: STb : EAST1:PAA:F4:F6	2
6	LT: STa : STb: EAST1:PAA:F4	19
6	LT: STb : EAST1:PAA: AIDA-I:F4	1
5	LT: STb : EAST1:F4:F6	1
5	LT: STa : STb:F4:F6	1
5	LT: STa : STb: EAST1:PAA	1
4	STb: EAST1:AIDA-I:F4	1
4	STb: EAST1:F4:F6	1
4	STa: STb : EAST1:F4	1
4	LT: STb : EAST1:F4	1
4	LT: STa : STb: EAST1	1
3	STa:F4:F18	1
3	STb: EAST1:F4	2
3	LT: STb : EAST1	1
3	STa: Stx2:Stx2e	1
2	STa:F6	1
2	LT: EAST1	1
2	EAST1:PAA	1
2	AIDA-I:intimin	1
2	EAST1:intimin	1
1	PAA	1
1	EAST1	3
1	STb	3
1	F41	1
0	No VG detected	9
	Total:	58

F4, F5, F6, F18, F41, fimbrial adhesins; LT, termolabile enterotoxin; STa, STb, thermostable enterorotoxins; Stx1, Stx2, Stx2e, Shiga-toxins; EAST1, enteroaggregative thermostable enterotoxin; PAA, porcine attaching and effacing associated protein; AIDA-I, adhesin involved in diffuse adherence.

#### 3.2.2 Established virulence genes in *Escherichia coli* isolated from suckling and weaned piglets

Of the 58 tested *E. coli* strains isolated from the small intestines, 16 were from weaned piglets and 42 from suckling piglets. In the 16 *E. coli* strains from post-weaning piglets, a total of 34 virulence factor genes were detected, averaging 2.12 genes per strain. In pre-weaned piglets, 42 strains were screened, and a total of 173 virulence factor genes were detected, averaging 4.11 per strain – almost double that of weaned piglets (**Table 4**). The most common virotype in the study, LT: STa : STb: EAST1:PAA:F4, was found in 2 of 16 (12.5%) strains from weaned piglets and in 17 of 42 (40.47%) strains from pre-weaned piglets.

### 3.3 Necropsy findings

Of the collected piglets, 55 were submitted for necropsy: 38 suckling and 17 weaned piglets, aged 2–82 days. There were 27 female and 28 male piglets, with 22/55 (40.00%) showing evident diarrhea. The main necropsy findings included various forms of enteritis, gastroenteritis, and enterocolitis, present in 40/55 cases (72.73%). Pathological changes indicating septicemia were found in 7/55 cases (12.73%), various forms of pneumonia in 3/55 cases (5.45%), and volvulus in 2/55 cases (3.64%). There was one case each of liver rupture, polyserositis, and cystic renal dysplasia. A complete list of piglets with relevant data, including age, sex, presence or absence of diarrhea, and presumed cause of death, is presented in **Supplementary Table 3**.

#### 3.3.1 Pathological findings in the digestive tract

**Figure 2:**
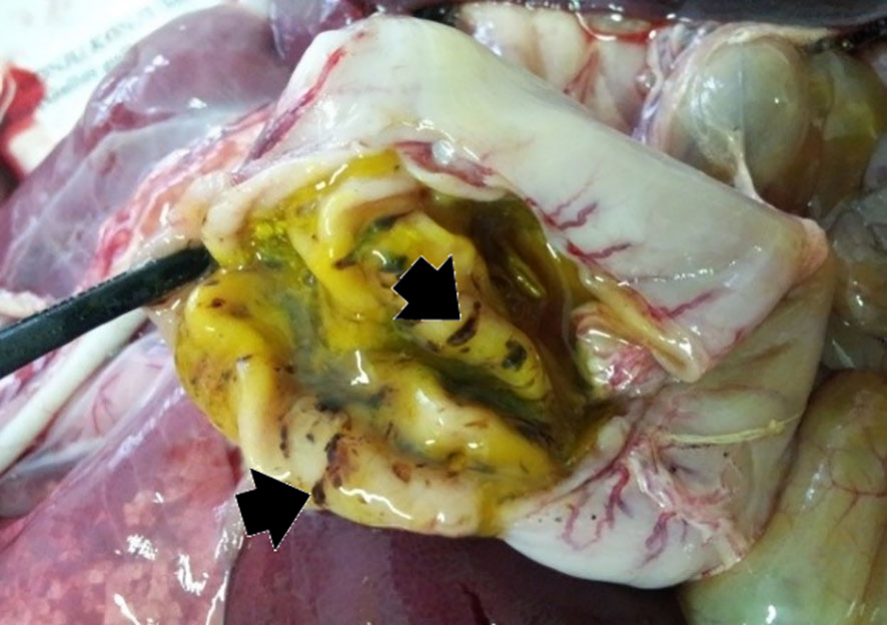
P55 – male, 35 days old, 6800 g. Stomach – multifocal, acute, mucosal hemorrhagic ulcers (arrows); abundant mucus, green content in the lumen corresponding bile regurgitation.

**Figure 3:**
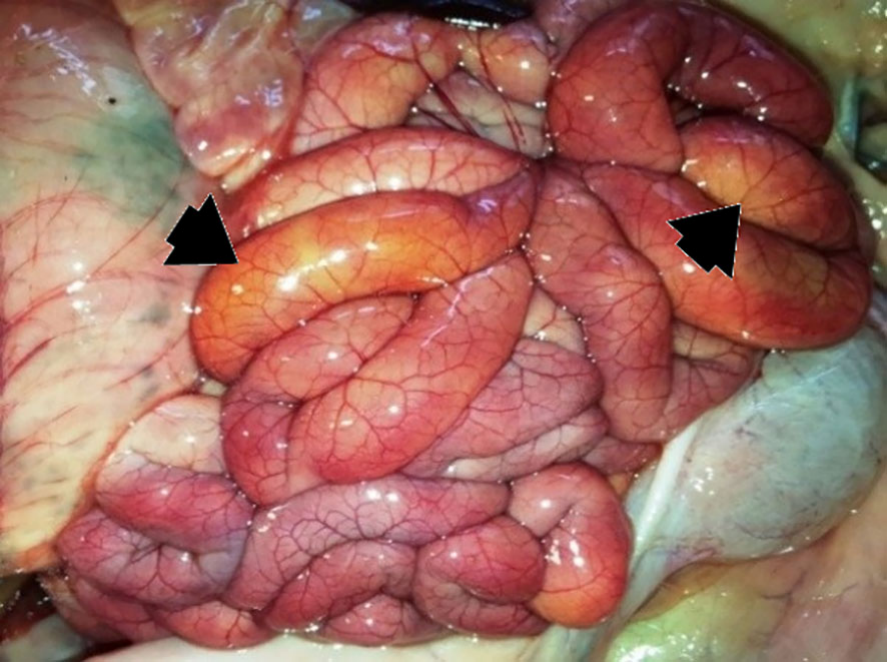
P52 – male, four days old, 1126 g. Dilated loops of jejunum and ileum filled with abundant yellow liquid content which is visible through the serosa (arrows). Marked hyperemia of the serosa and mesentery.

**Figure 4:**
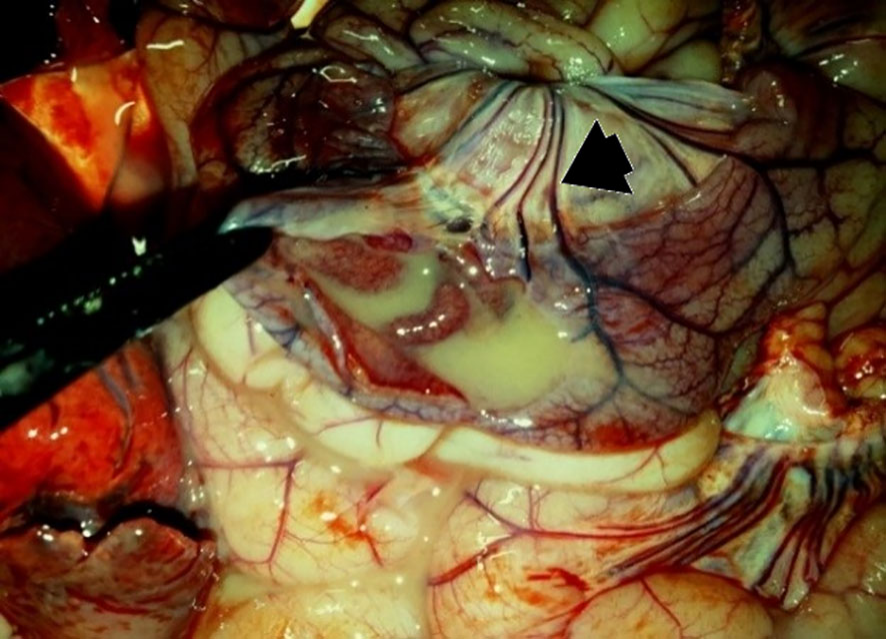
P51 – female, 55 days old, 7100 g. The lumen of the ileum contains yellow-gray liquid content. The mucosa is diffusely hyperemic with areas of small punctate hemorrhages. Hyperemia of the serosa and mesentery (arrow).

**Figure 5:**
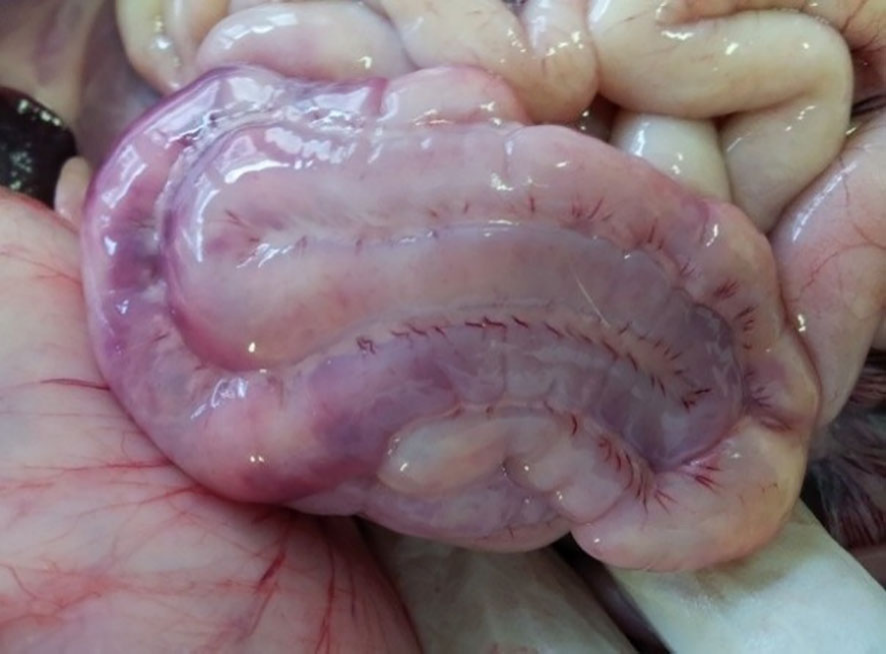
P42 – female, 13 days old, 2231 g. Spiral colon: edema of the mesocolon and serosa.

**Figure 6:**
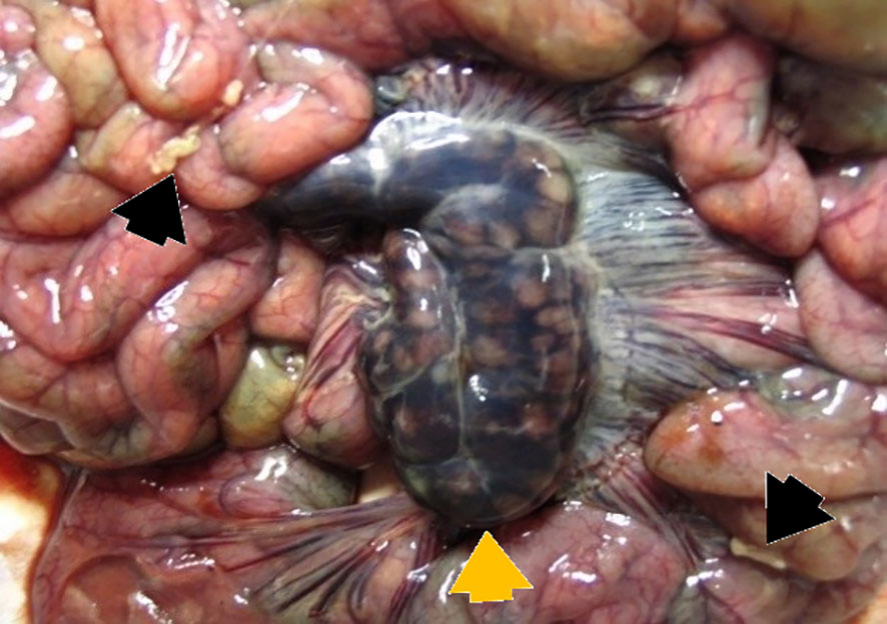
P4 – male, 26 days old, 5710 g. Subcapsular hemorrhage of the mesenteric lymph nodes with pronounced pseudomelanosis (black iron sulphide pigment; yellow arrow). Fibrin strands are found on the ileal loops (black arrow). Ileal serosa and the mesentery show marked hyperemia.

The stomach, small and large intestines, mesenteric lymph nodes, and mesentery - which are closely associated with the intestinal tract - are the primary sites of gross lesions in piglets with colibacillosis. Consequently, regions of the digestive tract cranial to the stomach were not described. Representative pathological findings in the digestive tract of affected piglets are shown in **Figures 2**–**6**.

#### 3.3.2 Gross pathological findings in the stomach

The most frequent gastric finding, observed in 13/55 of total cases (23.64%), was dilatation with retained contents; considering different categories (pre- and post-weaned piglets) – undigested caseous milk clots were found in 11/38 suckling piglets (28.95%), while undigested dry feed with yellowish fluid was present in 2/17 weanling piglets (11.76%). The gastric wall was often distended and thinned (12/55 cases, or 21.82%), with prominent hyperemia of the subserosal vasculature at the greater curvature. Disseminated acute hemorrhagic ulcers were found in the gastric mucosa in 8/55 cases (14.55%) (**Figure 2**), while chronic mucosal ulcers were observed in only 2/55 cases (3.64%). Lesions were consistently localized in the fundic glandular area, with no detectable changes in the cutaneous gastric mucosa of the pars oesophagea and pars pylorica in the examined piglets.

#### 3.3.3 Gross pathological findings in the small intestines

The ileum and jejunum were the most frequently affected segments of the small intestine in the examined piglets, while no significant macroscopic changes were observed in the duodenum. Of the 55 piglets reviewed, 16 (29.09%) had a dilated and flaccid small intestinal wall (**Figure 3**), and an equal number showed excessive mucus accumulation and hemorrhaging in the small intestinal mucosa. In nearly half of the piglets (26/55, 47.27%), the lumen of the small intestine was filled with liquid, yellow contents (**Figure 4**). In 11/55 (20.00%) of cases, the intestinal contents were frothy and ranged in color from dark yellow to orange. In 10/55 (18.18%) piglets, the small intestine was empty but contained abundant mucus, while in seven animals, significant amounts of blood were present in the small intestinal contents.

#### 3.3.4 Gross pathological findings in the large intestine

Compared to the small intestine, gross lesions in the colon were observed less frequently. The most common findings included subserosal and mesocolonic edema (17/55 cases, or 30,91%), particularly in the region of the spiral colon (**Figure 5**). In 19 of the 55 piglets examined (34.54%), the colonic contents were fluid, while in eight piglets (14.55%), visible blood was present. Diffuse mucosal hyperemia, accompanied by abundant mucus in the luminal contents, was noted in five animals (9,09%).

#### 3.3.5 Gross pathological findings in the mesentery lymph nodes

Macroscopic changes in the mesenteric lymph nodes were observed in a total of 20/55 piglets (36.36%). Enlargement of the mesenteric lymph nodes with pronounced lymphatic congestion was found in 10/55 animals (18.18%) while subcapsular hemorrhages were observed in 10/55 cases (18.18%) (**Figure 6**).

### 3.4 Microscopic morphological findings

We assessed 55 piglet carcasses as suitable for testing. All were submitted for necropsy, but only organs from *E. coli*+/VG+ piglets were processed for histology. Therefore, tissues from 46 piglets in which VGs were detected in the isolated *E. coli* strains were processed for histopathological examination, but in several cases, histological samples were not evaluated due to poor sample quality. Histological sections were prepared from a total of 195 organs and tissues: 39 stomach (STO) samples, 45 small intestine (SMI) samples, 40 colon (LAI) samples, 31 mesentery (MES) samples, and 40 mesenteric lymph nodes (MLN) samples. Intensity of histological lesions in organs and average pathoscores according to *E. coli* pathotypes are shown in **Table 6**. The average lesion scores ranged from 1.94 in the mesentery to 3.80 in the small intestine, which exhibited the most pronounced histopathological changes. Microscopic examination revealed significantly greater tissue damage in the small intestine and mesenteric lymph nodes compared to the stomach and mesentery, and moderately more severe damage compared to the colon. The grade of microscopic lesions in organs and the average pathoscore for each piglet are presented in **Supplementary Table 4**.

**Table 6:** Intensity of histological lesions in organs and average patoscore according to *Escherichia coli* pathotype.

Average grade of microscopic lesions in piglets (0-4)					
ORGAN	SMI	MLN	LAI	STO	MES
Average	3,80	3,10	2,90	2,33	1,94
Average patoscore according to pathotype					
Enterotoxigenic/Shiga-toxigenic *E. coli* (ETEC/STEC) hybrid					3,25
Enterotoxigenic *E. coli* (ETEC)					2,98
Enteropathogenic *E. coli* (EPEC)					2,75
Non-specific virotypes					2,70

SMI, small intestines; LAI, large intestines; MLN, mesentery lymph nodes; STO, stomach; MES, mesentery.

#### 3.4.1 Microscopic morphological changes in the stomach

**Figure 7:**
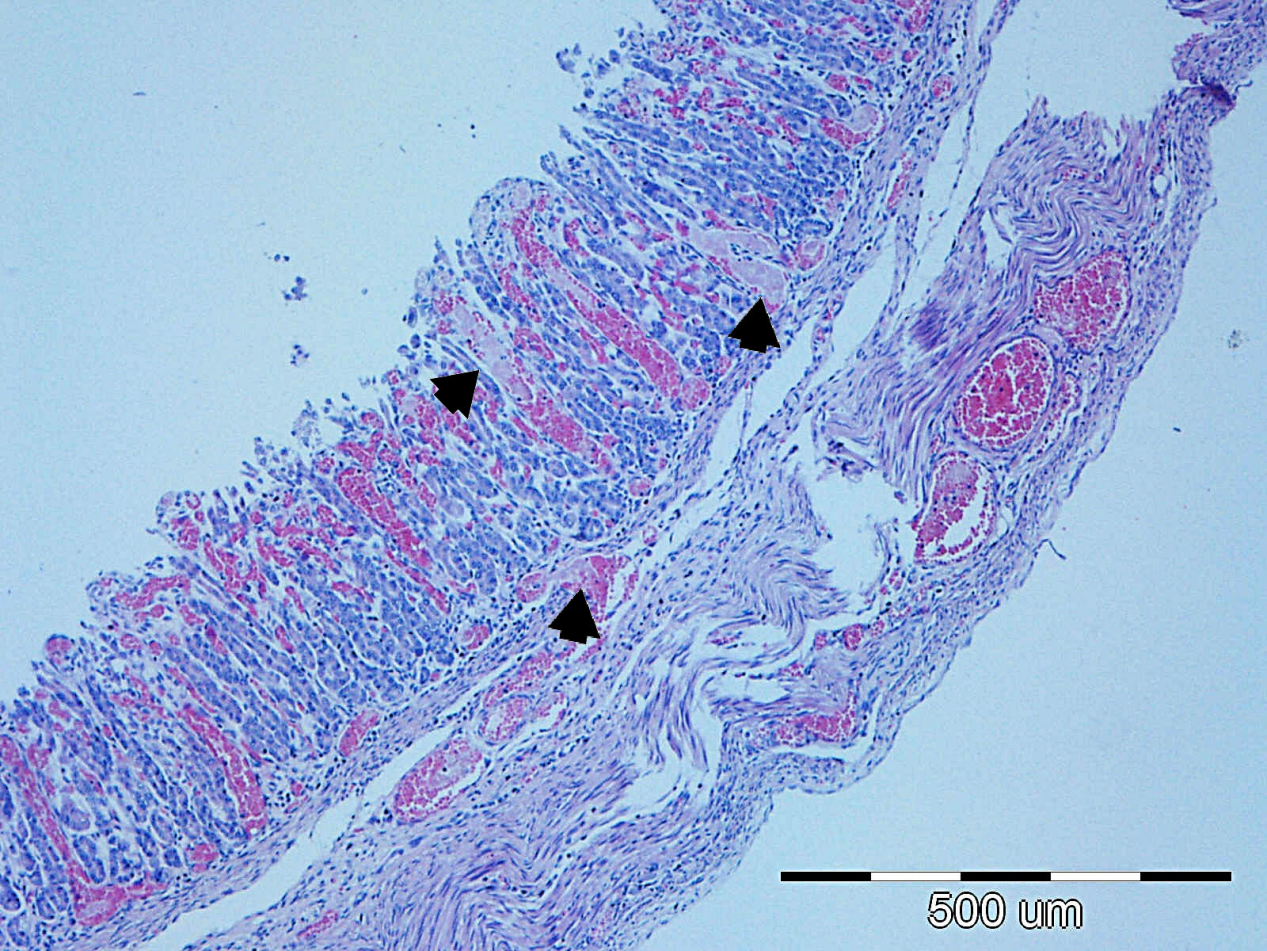
P28 Stomach, longitudinal cut through the wall, degree of damage 3: diffuse hyperemia in all layers, multifocal formation of fibrin thrombi (arrows) in the lamina propria of the glandular mucosa and submucosa. Hematoxylin and eosin stain (HE), objective magnification 10x.

**Figure 8:**
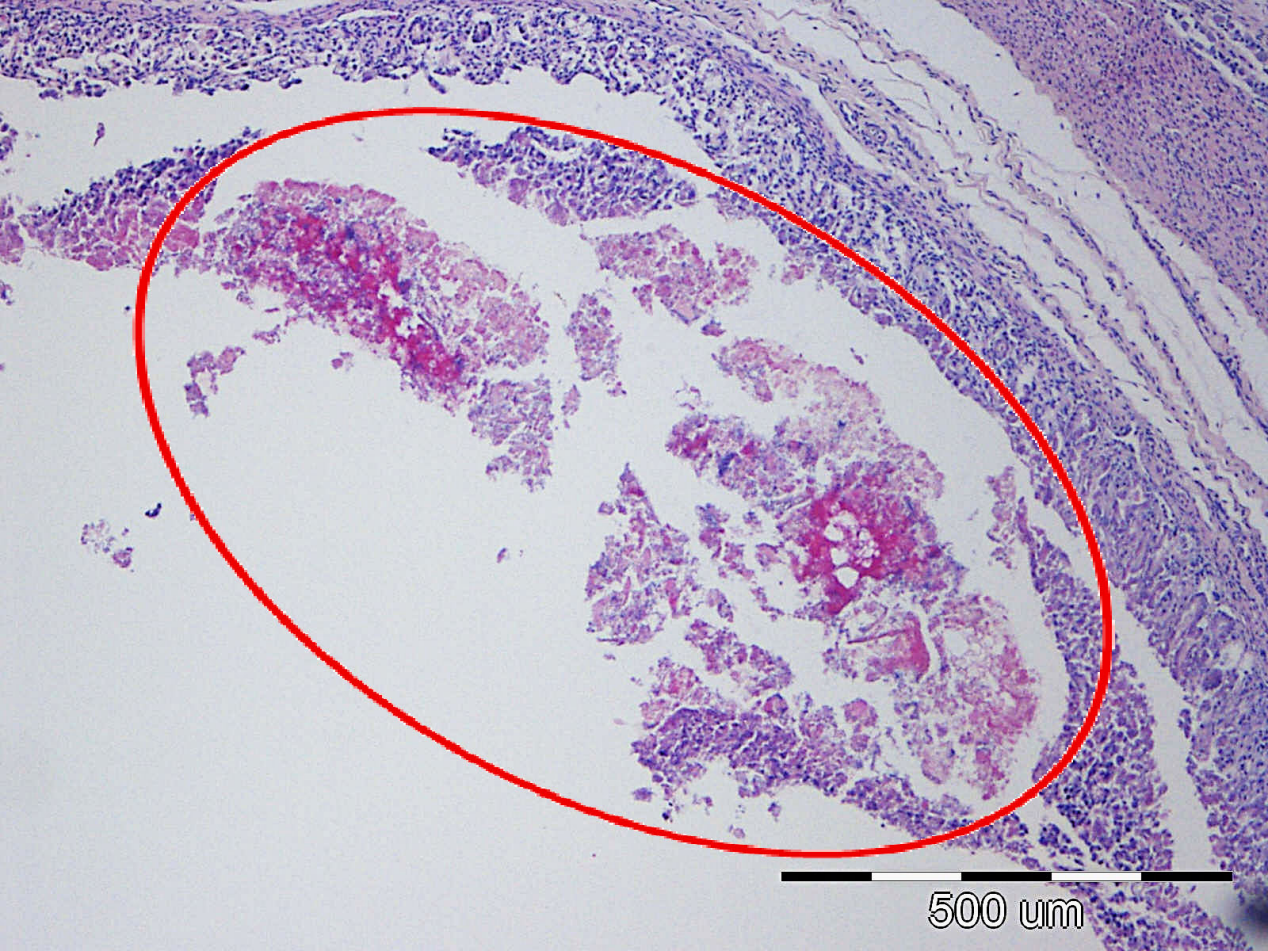
P47 Stomach, longitudinal cut through the wall, degree of damage 4: focal necrosis in the mucosa (circle). Necrotic area is surrounded by neutrophils; parietal glands show atrophy, the mucosal lamina propria is infiltrated by neutrophils. HE, 10x.

The mildest gastric lesions (grade 1) included focal atrophy of the tubular glands, mild subserosal edema, moderate hypertrophy of the muscularis layer, and mild hyperplasia of the foveolar cells on the mucosal surface. The most frequently observed change was diffuse hyperemia of the gastric wall (grade 2), which was found in 21 of 39 (53.84%) stomachs examined. Of the 21 cases, in 11 piglets, hyperemia was the result of death with a full stomach. In contrast, in 10 individuals the stomach was empty and contained some mucus or a smaller amount of watery content. Lesions classified as grade 3 were characterized by intense bacterial colonization of the superficial mucus, fibrin thrombi within mucosal blood vessels, and neutrophilic infiltration in the lamina propria of the gastric mucosa, accompanied by marked intravascular leukocytostasis. The most severe histological damage (grade 4) was characterized by mixed or purulent vasculitis affecting blood vessels of the muscularis and serosa, occlusive thrombi in all layers of the stomach wall (**Figure 7**), mucosal erosions and ulcers, and hemorrhagic-purulent and/or necrotic gastritis (**Figure 8**).

#### 3.4.2 Microscopic morphological changes in the small intestine

Based on gross pathological findings, grade 4 lesions were observed in the microscopically examined segments of the jejunum and ileum in 38/45 (84.44%) of cases. Lesion grades in the small intestine, along with their corresponding frequencies, are presented in **Table 7**.

**Table 7:** Grading and distribution of histological lesions in intestines and mesenteric lymph nodes.

Value	Small intestine		%
0	Absence of lesions		0,00
1	JEJUNUM: Edema in the lamina propria of the submucosa, dilation of the lymphatic vessels	ILEUM: Edema in the lamina propria of the submucosa, changes in Peyer’s patches (hyperplasia, histiocytosis, depletion)	0,00
2	Hyperemia of the wall, inflammatory infiltrate with an increased number of plasma cells and lymphocytes or mononuclear cells and globular lymphocytes, goblet cell hyperplasia, increased cellularity of the lamina propria of the mucosa, reduced colonization of brush borders		4,45
3	Wall distension, villous shortening, increased mucus secretion, hemorrhage, early thrombus formation, microabscesses		11,11
4	Hemorrhagic infarctions, thromboses accompanied by atrophy or necrosis of the mucosa, loss and desquamation of the epithelium, inflammatory infiltrates predominantly composed of neutrophils, bacterial colonization of the brush borders and enterocytes, as well as of the blood vessels in the lamina propria and submucosa		84,44
Value	Large intestine/colon		%
0	Absence of lesions		5,00
1	Mucosal hypertrophy		0,00
2	Edema and hyperemia of the wall, goblet cell hyperplasia, mononuclear inflammatory infiltrate		22,50
3	Bacterial colonization of enterocytes, surface mucus, cellular debris, and crypt lumens; mucus hypersecretion; wall distension; hemorrhages; microabscesses; shortening and fusion of villi; mixed inflammatory infiltrate		45,00
4	Bacterial colonization in the lamina propria of the submucosa and blood vessels; thrombosis and hemorrhages with occlusive thrombi; epithelial desquamation and mucosal erosions; inflammatory infiltrate dominated by neutrophils; epithelial necrosis of intestinal villi		27,50
Value	Mesentery lymph nodes		%
0	Absence of lesions		0,00
1	Edema (lymphatic stasis), depletion – follicular histiocytosis and lymphocyte pyknosis/reactive lymphocyte hyperplasia		12,50
2	Hyperemia, increased number of globular lymphocytes		2,50
3	Histiocytosis of the medullary and cortical sinuses, infiltration of eosinophilic granulocytes, nodal intravascular neutrophilic leukostasis, leukostasis in perinodal lymphatic vessels, fibrin thrombi		47,50
4	Neutrophilic infiltration of the lymph node, microabscesses, subcortical purulent lymphadenitis, granulomatous lymphadenitis, bacterial colonization		37,50

##### 3.4.2.1 Microscopic morphological changes in the jejunum

**Figure 9:**
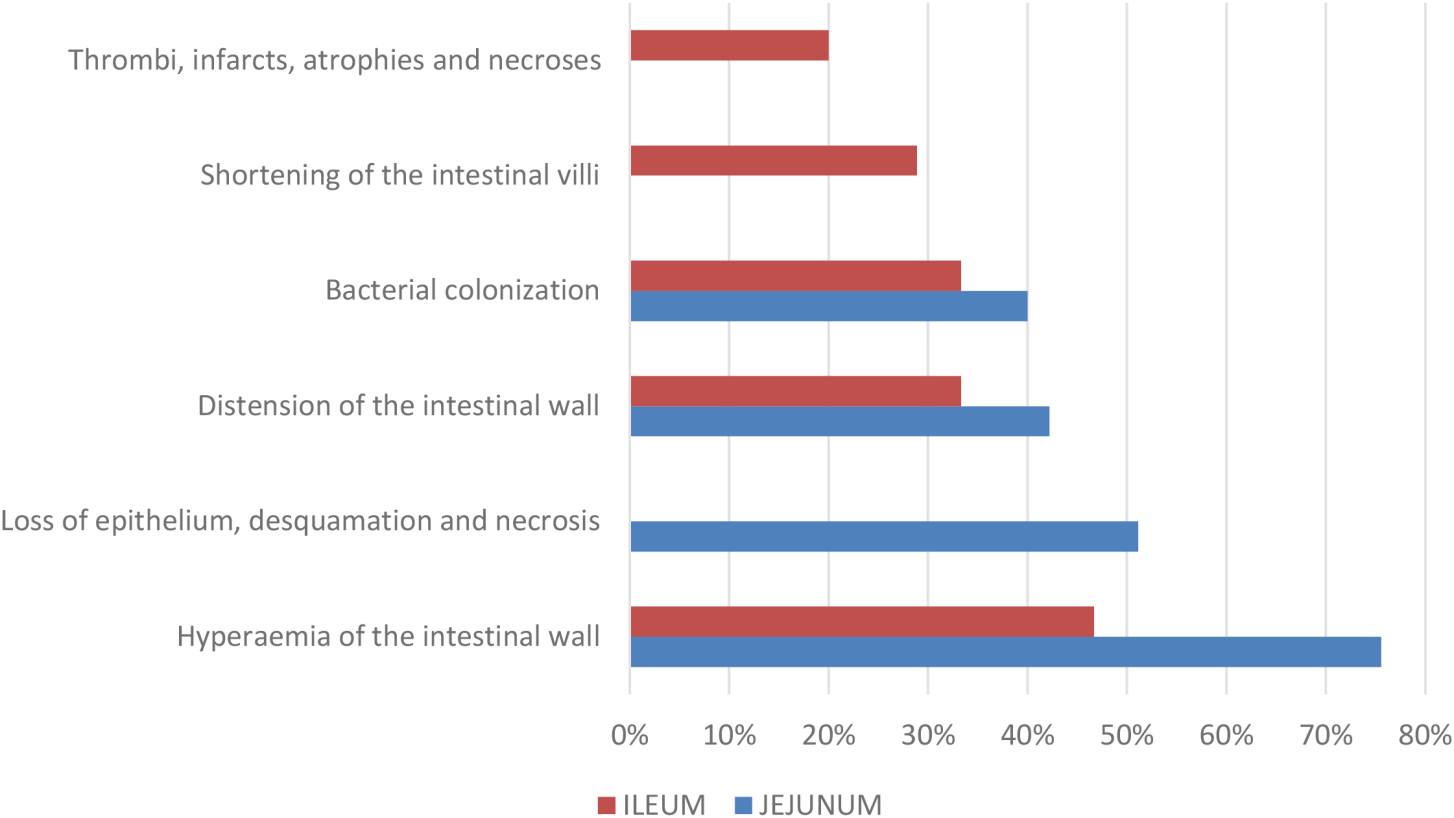
The most common lesions of the small intestine.

**Figure 10:**
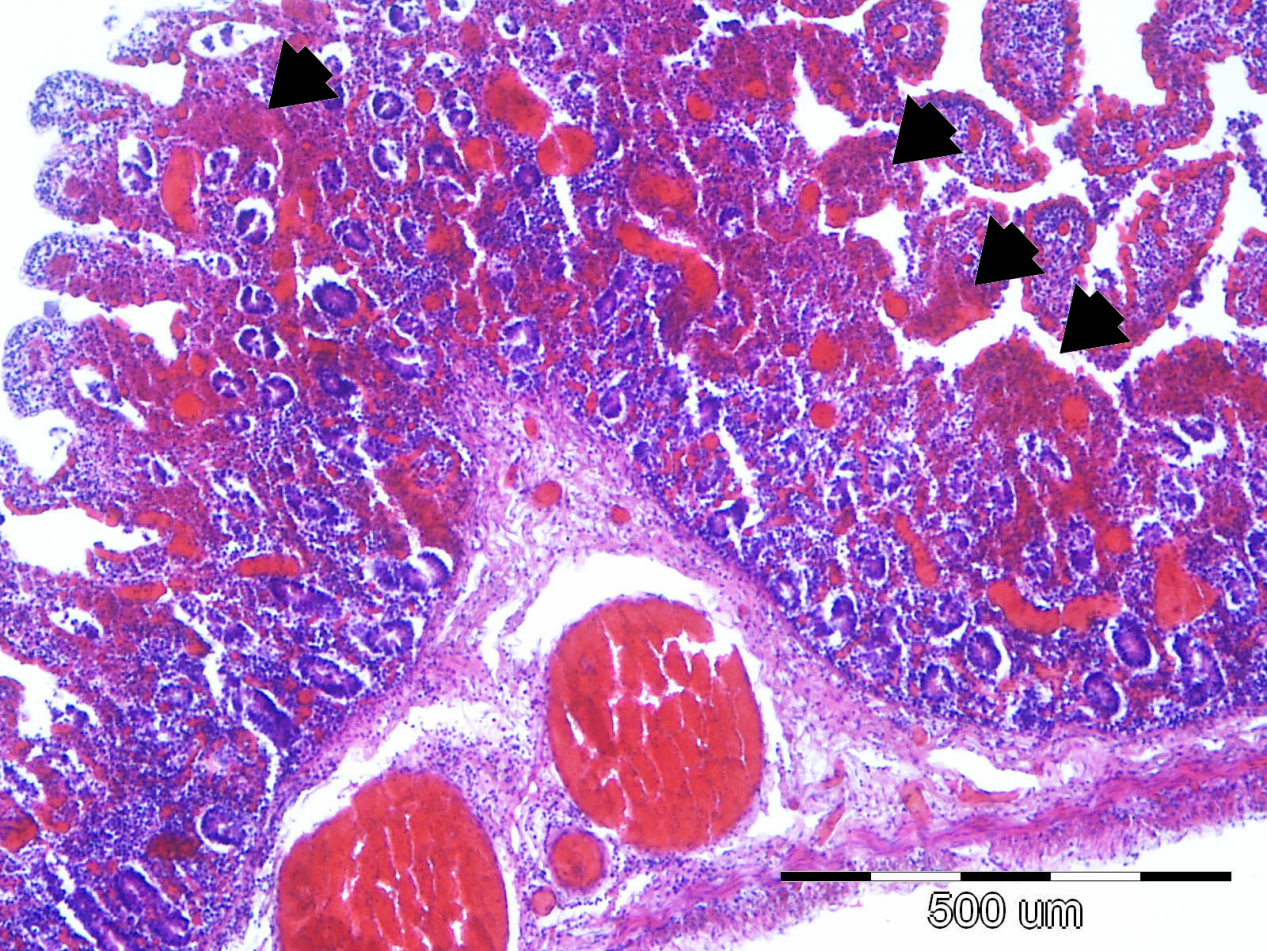
P6 Jejunum, longitudinal cut, degree of damage 4: diffuse congestion with dilation of blood vessels, acute hemorrhage (arrow) in the mucosal lamina propria; HE, 10x.

**Figure 11:**
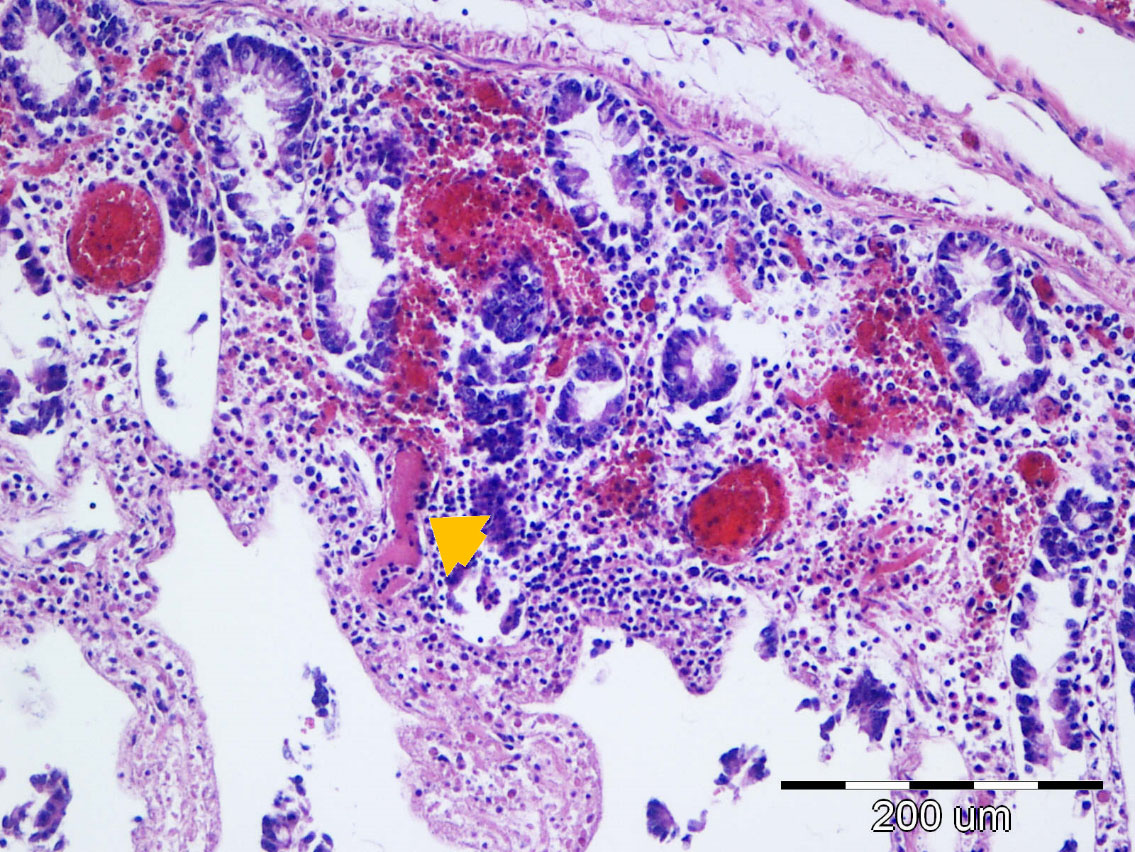
P34 Jejunum, longitudinal cut through the mucosa and submucosa, degree of damage 4: necrotic purulent jejunitis; diffuse hyperemia, fibrin thrombosis (arrow) resulting in villous necrosis; multifocal acute hemorrhage; HE, 20x.

In the sections of the jejunum examined, the most frequently observed changes were wall hyperemia, epithelial loss, desquamation and necrosis, distension of the intestinal wall, and bacterial colonization (**Figure 9**), which occurred most frequently in the superficial mucus and brush borders of the enterocytes, but also in the blood vessels and lamina propria. The term distension refers to maximum stretching of the intestinal wall and thinning of the mucosa as a result of atony and a considerable accumulation of fluid and gas in the intestine. The highest-grade jejunal lesions are shown in **Figures 10**, **11**.

##### 3.4.2.2 Microscopic morphological changes in the ileum

**Figure 12:**
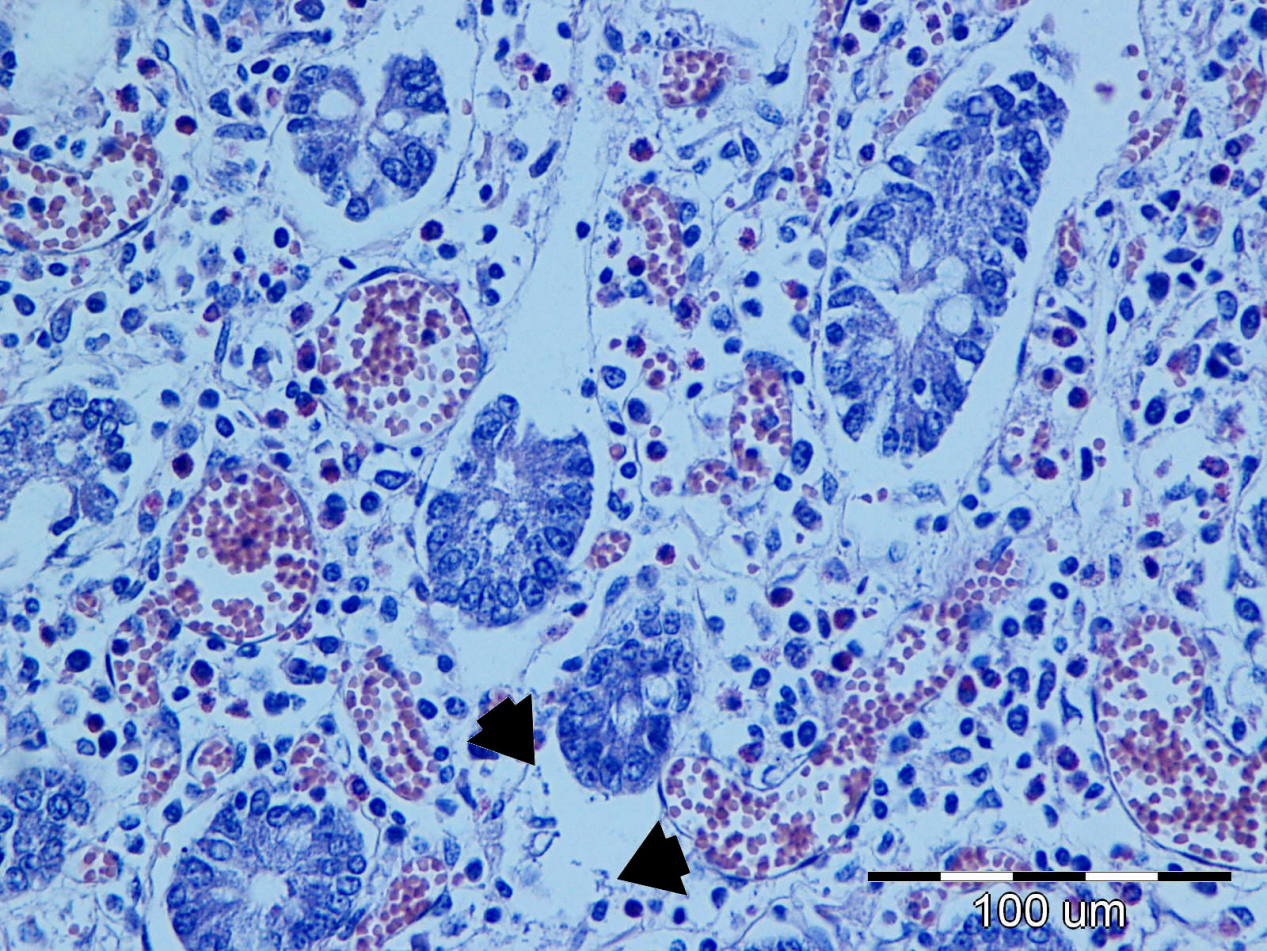
P6 Ileum, cross-section through the mucosa, degree of damage 4: diffuse edema of the lamina propria, moderate neutrophilic inflammation, hyperemia and focal bacterial penetration (arrows); HE, 40x.

**Figure 13:**
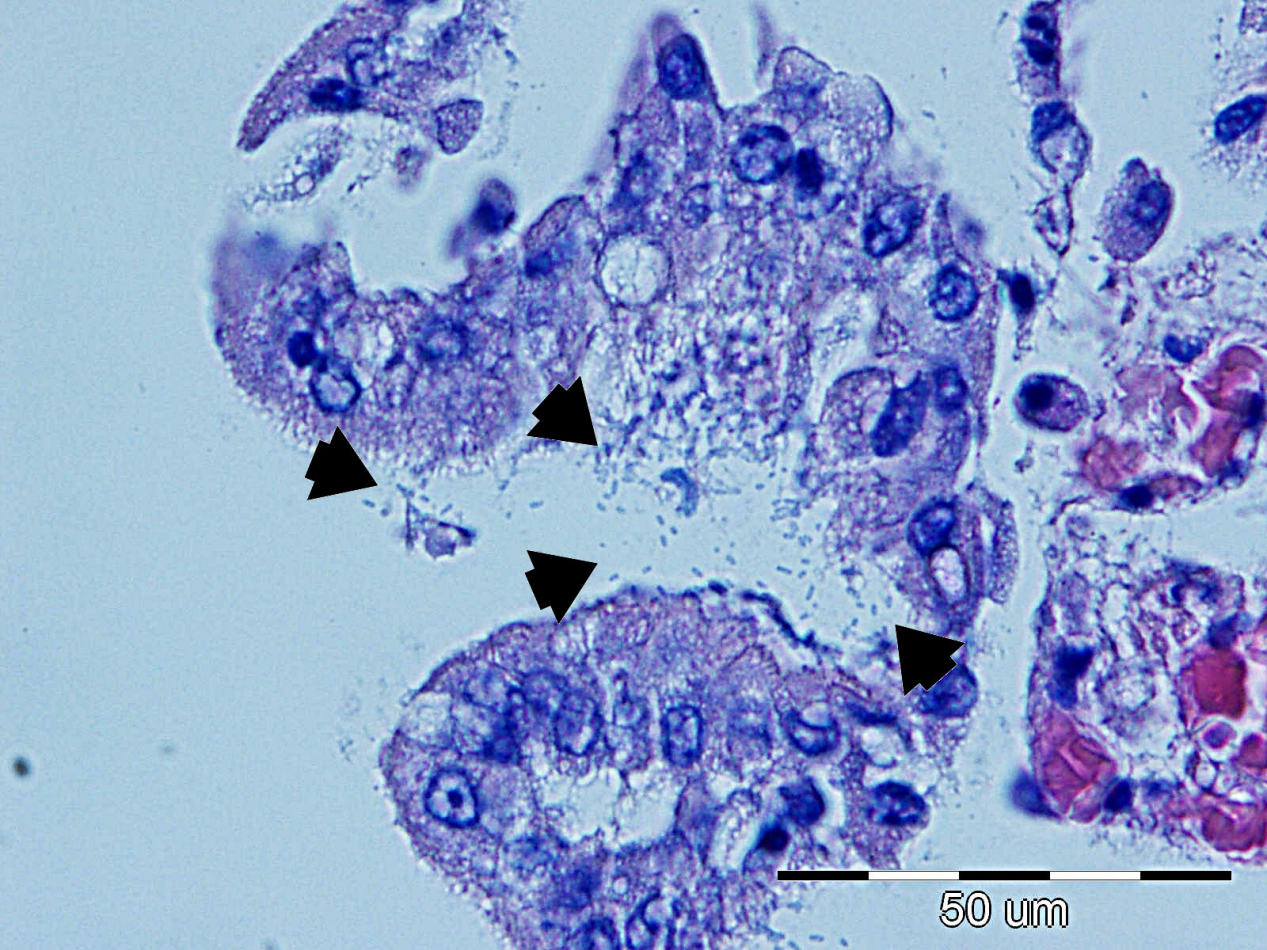
P28 Ileum, longitudinal cut through the tip of the villus, degree of damage 4: dilation and hyperemia of the blood vessels of the lamina propria, abundant short bacilli in the lumen (arrows); HE, 100x.

Representative lesions of the ileum are shown in **Figure 9**. The most frequent finding was hyperemia of the intestinal wall layers, observed in 31/45 cases (68.89%), followed by wall distension in 22/45 cases (48.89%). Bacterial colonization was most often observed on the brush border of enterocytes (12/45 cases, 26.67%), but was also detected in blood vessels of the lamina propria (7/45 cases, 15.56%) and within the superficial mucus (3/45 cases 6.67%). Shortening of intestinal villi was typically accompanied by wall distension and was occasionally associated with inflammatory infiltration in the lamina propria (6/45 cases, 13.33%). These lesions were rarely observed in isolation: hyperemia co-occurred with wall distension in 10 cases, with bacterial colonization in 11 cases, and with villous shortening in 10 cases. In seven piglets, hyperemia, distension, and villous shortening were present simultaneously. In five cases, these lesions were further accompanied by epithelial loss or mucosal necrosis (**Figures 12**, **13**). Histopathological changes in Peyer’s patches varied with age. In neonates, lesions included hyperplasia with infiltration of globular lymphocytes, whereas in weaned piglets, Peyer’s patches showed signs of lymphoid depletion, often accompanied by histiocytosis and/or marked pyknosis in follicular areas. These changes were observed in 3/34 (8.82%) of pre-weaning piglets and 2/12 (16.67%) of weaned piglets.

#### 3.4.3 Microscopic morphological changes in the large intestine

**Figure 14:**
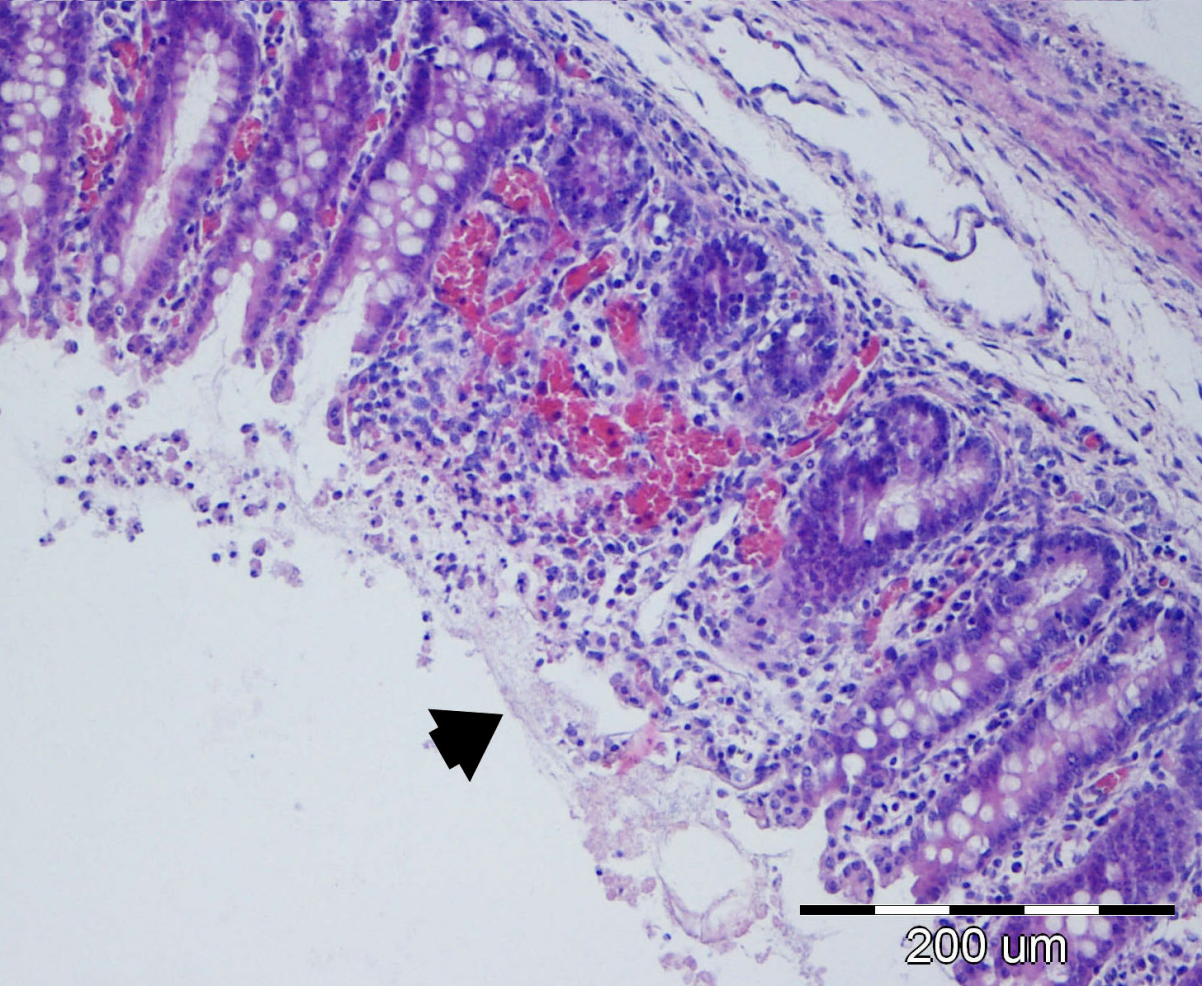
P41 Colon, longitudinal cut, degree of damage 4: erosive colitis, necrosis of villi tips (arrow), hyperemia and minor perivascular hemorrhages, edema and dilatation of lymphatic vessels of the submucosal lamina propria, moderate inflammation of mucosal lamina propria; HE, 20x.

**Figure 15:**
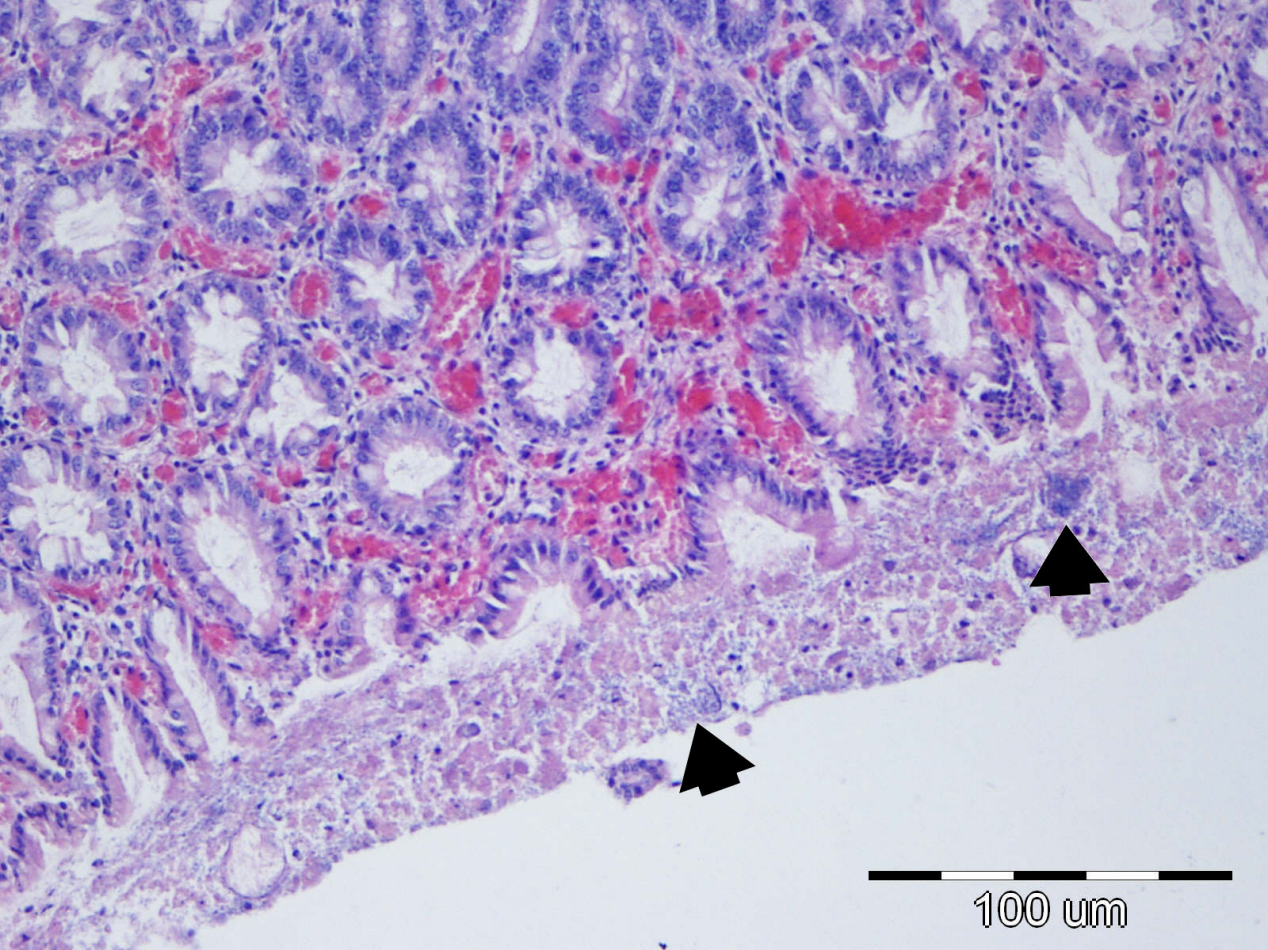
P42 Colon, cross-section through the mucosa, degree of damage 4: diffuse hyperproduction of mucus, with bacterial colonies (arrows) and cellular detritus present within the mucus; HE, 40x.

Edema and hyperemia of the colonic wall were the most frequent findings, observed in 25/40 (62.50%) of examined tissue sections. Bacterial colonization of enterocytes, surface mucus, cellular debris, or crypt lumina was identified in 21/40 (52.50%) of cases, whereas vascular colonization within the lamina propria of the submucosa was observed in only 3/40 (7.50%) of piglets (**Figures 14**, **15**). Colonic histological lesions, along with their corresponding scores and prevalence, are presented in **Table 7**.

#### 3.4.4 Microscopic morphological changes in the mesentery

**Figure 16:**
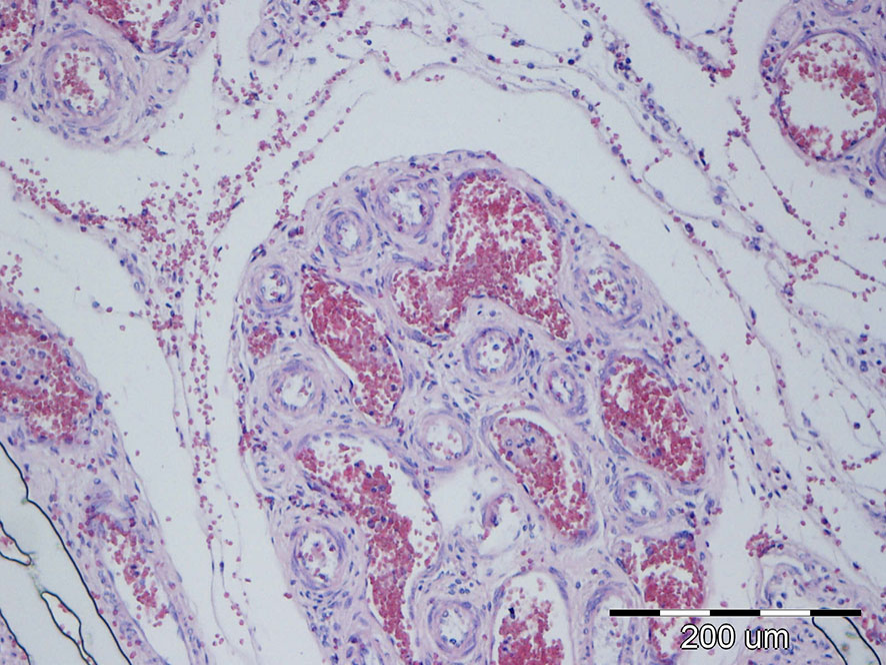
P40 Mesocolon, cross-section through blood vessels, degree of damage 3: severe edema, hyperemia and mild perivascular hemorrhage. HE, 40x.

Microscopic lesions of the mesentery, graded by severity, included the following: 1) hyperemia; 2) acute hyperemia with perivascular edema and intravascular hemolysis; 3) intravascular leukocytostasis with hemorrhage; and 4) fibrin thrombi (**Figure 16**).

#### 3.4.5 Microscopic morphological changes in the mesentery lymph nodes

**Figure 17:**
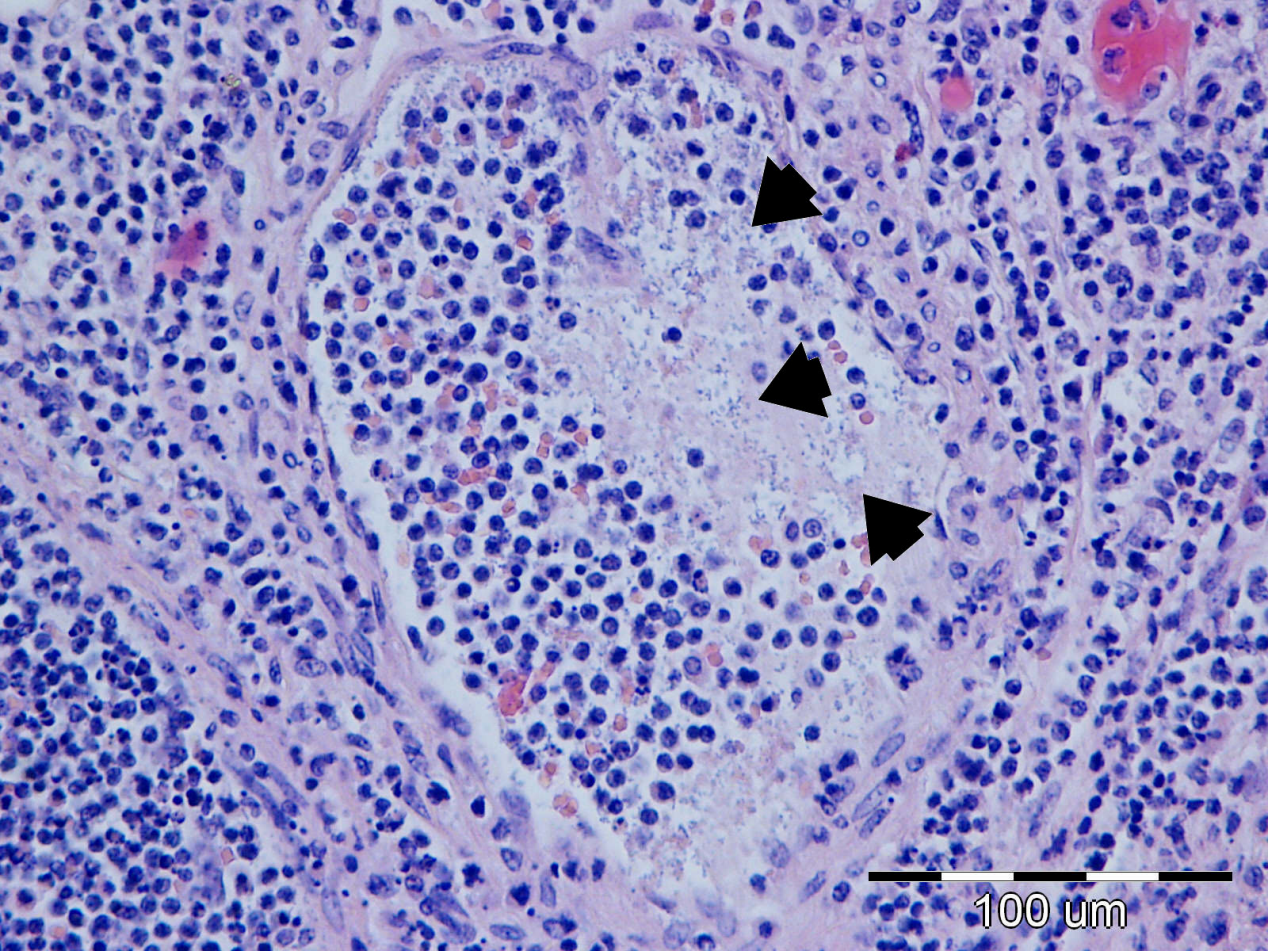
P37 Mesenteric lymph node, degree of damage 4, purulent lymphadenitis: neutrophilic intravascular leukocytostasis in medular blood vessels and intravascular bacterial colonization (arrow). HE, 40x.

According to the established histopathological criteria, mesenteric lymph nodes exhibited the second-highest average lesion score (3.10), following the small intestine. The most frequently observed lesions included histiocytosis of the medullary and cortical sinuses, along with hyperemia. Among the highest-grade lesions (grade 4), which were observed simultaneously in some cases, microabscesses were identified in 11/40 (27.50%) of cases, purulent lymphadenitis (**Figure 17**) in 4/40 cases (10.00%), bacterial colonization in 3/40 cases (14.16%), and both granulomatous lymphadenitis and neutrophilic infiltration in 2/40 (5.00%) cases each. A summary of lesion categories and their prevalence is presented in **Table 7**.

#### 3.4.6 Severity of histological lesions and pathoscore across *Escherichia coli* pathotypes

The most severe histopathological changes in piglets infected with ETEC were observed in the small intestine and mesenteric lymph nodes. The lowest cumulative pathoscore was recorded in piglets infected with the non-specific virotypes, strains that cannot be assigned to a recognized diarrheagenic pathotype because they possess none of the hallmark VGs. The highest average score (3.25) was associated with ETEC: STEC hybrid pathotype. Pathoscores by pathotype are presented in **Table 6**.

### 3.5 Results of the statistical analysis

Piglets P27, P37, P45, and P47 were each found to carry two *E. coli* strains, with differing virulence gene profiles identified within the same animal. To ensure accurate assessment of the relationship between virulence genes and pathomorphological changes, both strains from each piglet were considered together and treated as a single finding in subsequent analyses.

A strong and statistically significant correlation was observed between the isolation of individual *E. coli* strains from the small and large intestine, indicating that these isolations were not independent events (Kendall’s Tau-b = 0.923, p = 0.0003).

The presence of the *faeG* (F4) gene in 32 *E. coli* strains was statistically associated with lesions in the small intestine and mesenteric lymph nodes. Similarly, the *eltA* (LT) gene, detected in 28 strains, was associated with lesions in both the small and large intestines as well as the mesenteric lymph nodes. The *estII* (STb) gene, present in 37 strains, was significantly associated with lesions in the small and large intestines. The *astA* (EAST1) gene, found in 39 isolates, was associated with lesions in the small intestine. In contrast, the *paa* (PAA) gene, detected in 26 *E. coli* strains, was linked to changes in the small and large intestines and mesenteric lymph nodes, while the *aidA* (AIDA-I) gene showed a statistically significant association with lesions in the stomach. Detailed associations between virulence genes and pathological changes are presented in **Table 8**.

**Table 8:** Association of virulence genes with pathological lesions (p<0,05).

VF gene	F4 *faeG*	F5 *fanC*	F6 *fasA*	F18 *fedA*	F41 *f41*	LT *eltA*	STa *estI*	STb *estII*	Stx1 *stx1*	Stx2 *stx2*	Stx2e *stx2e*	EAST1 *astA*	intimin *eae*	PAA *paa*	AIDA-I *aidA*
n	32	0	7	1	1	28	29	37	0	1	1	39	2	26	3
SMI	0,003					0,002		0,001				<0,001		0,002	
LAI						0,017		0,026						0,047	
MLN	0,049					0,030								0,03	
MES															
STO															0,019

VF, virulence factor; F4, F5, F6, F18, F41, fimbrial adhesins; LT, termolabile enterotoxin; STa, STb, thermostable enterorotoxins; Stx1, Stx2, Stx2e, Shiga-toxins; EAST1, enteroaggregative thermostable enterotoxin; PAA, porcine attaching and effacing associated protein; AIDA-I, adhesin involved in diffuse adherence; SMI, small intestines; LAI, large intestines; MLN, mesentery lymph nodes; STO, stomach; MES, mesentery.

The cumulative pathoscore, reflecting the total severity of pathological changes, differed significantly between suckling and weaned piglets (p = 0.0091), as well as between piglets with and without diarrhea (p = 0.0223). Lesions were more severe in suckling piglets and in those presenting with diarrhea.

Statistically significant differences in pathoscore were also associated with the presence of specific virulence genes: *faeG* (p = 0.0001), *eltA* (p = 0.0001), *estII* (p = 0.0001), *paa* (p = 0.0002), and *astA* (p = 0.0029). In all cases where a statistically significant difference was found, the median values were higher if the bacterial isolate carried the respective genes.

### 3.6 Practical diagnostic implications based on research findings

Based on integrated data from bacteriological and molecular tests, as well as histopathological findings, it is possible to link proven virulence genes with tissue injuries and draw practical conclusions and diagnostic recommendations. The correlation between virulence genes and lesions in piglets with suspected colibacillosis, with practical diagnostic implications, is summarized in **Table 9**.

**Table 9:** Correlation between *Escherichia coli* virulence genes and lesions in piglets: practical diagnostic implications.

Virulence gene	Associated lesions	Tissue(s) involved	Notes based on study findings
*faeG* (F4)	Severe epithelial damage Brush-border colonization Higher cumulative lesion scores	Small intestine (jejunum, ileum); also large intestine	Strongest association with high lesion severity; consistent with dense mucosal adherence.
*eltA* (LT)	Epithelial injury Fluid-secretion–associated mucosal changes Increased histopathology scores	Small and large intestine	One of the most significant VGs associated with severe intestinal lesions.
*estII* (STb)	More severe mucosal lesions Crypt and epithelial involvement	Small intestine; colon	Significantly associated with higher histopathological scores.
*astA* (EAST1)	Severe histological changes Hyperemia, edema, mucosal damage	Small intestine; colon; mesenteric lymph nodes	Part of the predominant virotype; most prevalent VF and linked with broader lesion distribution.
*paa* (PAA)	Intestinal mucosal lesions Lymphoid depletion/inflammatory changes	Small intestine; large intestine; mesenteric lymph nodes	Only VG explicitly linked to both intestinal and lymph-node lesions, suggesting deeper or more systemic involvement.
*aidA* (AIDA-1)	Gastric erosions/lesions	Stomach	The *only* VG associated with gastric pathology.
*estI* (STa)	Contributed to lesion severity when part of hybrid virotypes	Mainly small intestine	Not individually mapped, but STa-containing virotype (e.g., STa: Stx2:Stx2e) had highest overall lesion severity.
*stx2/stx2e* (Stx/Stx2e)	Part of high-severity lesion profiles in hybrid ETEC: STEC strains	Small intestine; colon	Associated with highest average pathoscore, especially when co-present with ETEC toxins.

F4, fimbrial adhesin; LT, termolabile enterotoxin; STa, STb, thermostable enterorotoxins; EAST1, enteroaggregative thermostable enterotoxin; PAA, porcine attaching and effacing associated protein; AIDA-I, adhesin involved in diffuse adherence; Stx2, Stx2e, Shiga-toxins.

## 4 Discussion

This study provides a detailed insight into the complex interplay between *E. coli* virulence gene profiles and the severity and distribution of gastrointestinal lesions in piglets affected by colibacillosis. It correlates specific virulence factors (VF) with both gross and microscopic changes in the affected tissues and compares the prevalence of *E. coli* pathotypes in piglets before and after weaning, in order to show the relationship between *E. coli* virulence genes and the severity of pathological changes in the digestive system of piglets. Numerous studies have shown that *E. coli* strains are responsible for both intestinal (Luppi, 2017; Castro et al., 2022) and extraintestinal infections (Connelly et al., 2025), as well as mortality in piglets before and after weaning (Fairbrother and Nadeau, 2019). *E. coli* strains are able to horizontally exchange genetic material located on plasmids (Nemati et al., 2025), allowing commensal strains to acquire virulence genes through conjugation and transduction (Kelly et al., 2009; Penadés et al., 2015; Lerminiaux and Cameron, 2019; Naidoo and Zishiri, 2025) and thus transform into pathogenic strains. Depending on the virulence factors present, different *E. coli* pathotypes can cause diseases that differ in clinical presentation, course and pathological features (Vijtiuk et al., 1995; Zajacova et al., 2013; Abubakar et al., 2017). Here, we will discuss the results of our research and compare them with existing knowledge.

Among the 58 *E. coli* isolates analyzed, 49/58 (84.48%) of strains carried at least one VG. This percentage is higher compared to previous studies, which reported rates ranging from 55.33% (Li et al., 2020), 58.30% (Tusiime et al., 2024), 68.60% (Schierack et al., 2006), and 71.25% (De Lorenzo et al., 2018) to 79,00% (Vu-Khac et al., 2007). The slightly higher proportion of VG-positive samples in our study is likely due to the fact that the examined strains were derived exclusively from intestinal organs of deceased piglets with overt lesions, whereas some of the studies mentioned above also included strains isolated from both clinically ill and healthy pigs. Differences in the prevalence of VGs may also be influenced by the number of genes tested, which varies across studies.

The predominance of the ETEC pathotype (37/58 or 63.79%) aligns with its well-established role in neonatal and post-weaning diarrhea (Nagy and Fekete, 2005; Brown et al., 2007; Dubreuil et al., 2016; De Lorenzo et al., 2018; Li et al., 2024). Importantly, the majority of ETEC strains in our study carry virulence genes associated also with EAEC, EPEC, or STEC pathotypes. This trend toward hybridization corroborates findings from recent research indicating a growing prevalence of recombinant or mosaic *E. coli* strains (Robins-Browne et al., 2016; Paiva et al., 2025). These hybrid strains likely have enhanced adaptability, they are more virulent (Yang et al., 2019) and have broader pathogenic potential. This is supported by the finding of our study, where virotype STa: Stx2:Stx2e was associated with the most severe overall lesions, and in agreement with other studies whose results indicate that ETEC: STEC should be considered as a new emerging pathogen (Nammuang et al., 2024; Paiva et al., 2025). The most prevalent virotype in this study - LT: STa : STb: EAST1:PAA:F4 - was detected in 32.76% of all isolates, with a marked predominance in pre-weaned piglets. This virotype’s combination of multiple adhesins and toxins suggests a particularly aggressive pathogenic profile and may explain the higher lesion severity observed in this age group.

The average number of virulence genes per isolate was nearly double in pre-weaned piglets (4.11) compared to weaned piglets (2.12), indicating that suckling piglets are exposed to more virulent *E. coli* strains. Besides the evident exposure of suckling piglets to more virulent strains, another reason colibacillosis is more severe in younger piglets is their immature immune systems and limited intestinal colonization resistance (Do et al., 2006; Luppi, 2017). Statistically significant differences in cumulative pathoscore between pre- and post-weaning piglets support this interpretation and further highlight the importance of age-specific disease management strategies.

The presence of *faeG* (F4), *eltA* (LT), *estII* (STb), *paa* (PAA), and *astA* (EAST1) was statistically associated with more severe histopathological changes, particularly in the small intestine and mesenteric lymph nodes. These findings affirm previous studies emphasizing the pivotal role of these virulence factors in mucosal colonization, enterotoxin-mediated fluid secretion, and inflammatory injury (Dubreuil, 2008; Andrade et al., 2010; Barros et al., 2023).

Among these, *faeG* and *eltA* had the strongest associations with high lesion scores in both the small and large intestines. The role of *faeG* in facilitating bacterial adherence to the villous epithelium - especially in the ileum - is well documented (Cheng et al., 2006; Li et al., 2007; Yan et al., 2009; Sugiharto et al., 2012; Ikwap et al., 2016; Castro et al., 2022). Our results confirm the results of other study (Van Breda et al., 2017) and show that F4 is associated with colibacillosis both before and after weaning. Likewise, *eltA*, encoding the LT toxin which disrupts cyclic AMP signaling in enterocytes, leading to diarrhea and epithelial damage is recognized as most common (Yang et al., 2019) and significant VG in diarrheic piglets (Lin et al., 2022). The gene *paa* was linked to changes not only in the small and large intestines but also in the mesenteric lymph nodes, suggesting that this non-fimbrial adhesin may contribute to systemic dissemination or deeper tissue invasion. Interestingly, the *aidA* gene for non-fimbrial AIDA-1 adhesin was the only virulence gene associated with gastric lesions, underscoring the diversity of tissue-specific pathogenic mechanisms among *E. coli* strains. Some other studies also showed a high prevalence of AIDA-1 adhesin gene as well as a high prevalence of *astA*-EAST1 toxin gene (Kongsted et al., 2018; Li et al., 2020) and found a significant association between the presence of EAST1 and AIDA-I with diarrhea in piglets (Tusiime et al., 2024). The *astA* gene, which encodes the EAST1 toxin, was one of the first virulence factors identified in EAEC isolates. However, unlike *aggR* (Morin et al., 2013; Guerrieri et al., 2019), it is not exclusive to EAEC. It can be found on small plasmids and transposons, and due to this mobility, EAST1 is highly prevalent in porcine *E. coli* populations, especially in ETEC and non-pathogenic commensals. This is supported by our study, as we found it in 39/58 (67.24%) *E. coli* strains. Although EAST1 may contribute to pathogenicity, it cannot be used as a diagnostic marker for the EAEC pathotype. Currently, EAST1 is considered an accessory virulence factor, but not a defining one. Therefore, the EAST1 virotype, found in 3/58 (5.17%) of strains, is considered a non-specific virotype, along with six other virotypes in this study, where none of the hallmark genes are present.

Histologically, the most severe damage was observed in the small intestine (mean score 3.80), followed by the mesenteric lymph nodes (3.10), the colon (2.91) and the stomach (2.23). This pattern reflects the known pathogenesis of *E. coli*-induced diarrhea, which focuses on colonization and disruption of the small intestine (Janke et al., 1989; Sha et al., 2024). Microscopically, there were characteristic lesions such as villous atrophy, epithelial desquamation, crypt hyperplasia and bacterial colonization of the brush border, especially in the jejunum and ileum. In contrast to the study that identified villous atrophy as the predominant lesion (Sha et al., 2024), we found it in 6/45 intestinal samples (13.33%). In this study, bacterial colonization of the enterocyte brush border was much more common than villous atrophy. Hyperemia and distension were frequent accompanying symptoms, which often led to hemorrhage, necrosis or transmural edema. In the colon, crypts filled with mucus and leukocytes with epithelial erosion were common. Peyer’s patches lesions varied according to age, with lymphoid depletion occurring more frequently in weaned piglets, possibly due to the systemic effects of the toxins. The pathological changes described in the most common ETEC pathotype are more extensive than those described previously (Do et al., 2006; Brown et al., 2007) and are the result of the expression of the acquired genes *astA* and *paa*. While ETEC was the most prevalent and pathogenic pathotype, the hybrid pathotype ETEC: STEC yielded the highest average pathoscore (3.25), with lesions similar to ETEC: STEC in experimental infection (Baek et al., 2023), indicating synergistic or additive pathogenic effects when multiple pathotype-defining virulence genes coexist.

## 5 Conclusions

This study emphasizes the value of integrating bacteriological, molecular and histopathological data for the accurate diagnosis and treatment of colibacillosis. The findings highlight important practical implications for the diagnosis of colibacillosis in piglets, because most isolates carried at least one virulence gene, and recombinant or mosaic and hybrid ETEC strains were common. The strong associations between these genes - particularly *faeG*, *eltA*, *estII*, *paa*, and *astA* - and higher lesion scores indicate that virulence-gene detection can help anticipate disease severity and guide interventions.

Based on the observed associations between virulence genes and lesion patterns, the diagnosis and management of colibacillosis in piglets can be improved through three key approaches:

- Tissue Sampling and Lesion Assessment - necropsy should focus on the jejunum and ileum, with attention to the colon, lymph nodes, and stomach when small-intestinal lesions are ambiguous. Age-specific interpretation of lesions is important for accurate assessment, as suckling piglets typically exhibit more extensive epithelial injury, whereas weaned piglets more frequently show lymphoid depletion in Peyer’s patches.
- Virulence-Gene Detection - expanded virulence-gene screening, including adhesins (*faeG*, *aidA*, *paa*) and toxins (*eltA*, *STa*, *STb*, *EAST1*, *stx2*, *stx2e*) can improve detection of classical and hybrid *E. coli* strains. Identification of *faeG* or *eltA* should be considered strong evidence for ETEC involvement, whereas *paa* or *astA* may indicate deeper tissue involvement or systemic effects.
- Integrated Interpretation - virulence-gene results should be interpreted alongside pathological changes and piglet age. Combining lesion distribution with virulence-gene patterns improves diagnostic certainty and supports timely implementation of treatment, management changes, and possible inclusion of relevant virulence factors in autogenous vaccines.

## Data Availability

The raw data supporting the conclusions of this article will be made available by the authors, without undue reservation.

## References

[B1] Abubakar R. H. Madoroba E. Adenubi O. Morar-Leather D. Fasina F. O. (2017). Bacterial pathogens of pigs with particular reference to *Escherichia coli*: A systematic review and meta-analysis. J. Vet. Med. Anim. Health 9, 159–185. doi: 10.5897/JVMAH2017.0594

[B2] Andrade J. A. B. Freymuller E. Fagundes-Neto U. (2010). Pathophysiology of enteroaggregative *Escherichia coli* infection: an experimental model utilizing transmission electron microscopy. Arq Gastroenterol. 47, 306–312. doi: 10.1590/S0004-28032010000300018, PMID: 21140095

[B3] Baek K. H. Tangchang W. Choi E. J. Lee W. K. Lee K. H. Lee H. K. . (2023). Experimental infection of post-weaned pigs with F18-encoding enterotoxigenic and enterotoxigenic/shigatoxigenic *Escherichia coli* strain isolated from the diarrheic feces in Korea. Open veterinary J. 13, 705–714. doi: 10.5455/OVJ.2023.v13.i6.5, PMID: 37545702 PMC10399650

[B4] Barros M. M. Castro J. Araújo D. Campos A. M. Oliveira R. Silva S. . (2023). Swine colibacillosis: global epidemiologic and antimicrobial scenario. Antibiotics 12, 682. doi: 10.3390/antibiotics12040682, PMID: 37107044 PMC10135039

[B5] Boisen N. Østerlund M. T. Joensen K. G. Santiago A. E. Mandomando I. Cravioto A. . (2020). Redefining enteroaggregative *Escherichia coli* (EAEC): Genomic characterization of epidemiological EAEC strains. PloS Negl. Trop. Dis. 14, e0008613. doi: 10.1371/journal.pntd.0008613, PMID: 32898134 PMC7500659

[B6] Brown C. C. Baker D. C. Barker I. K. (2007). “ Escherichia coli,” in Pathology of domestic animals, 5th edition, vol. 2. (Philadelphia: Elsvier Saunders) 2 (1), 183–193.

[B7] Canal A. M. Cubillos V. Zamora J. Reinhardt G. Paredes E. Ildefonso R. . (1999). Histopathological lesions in the small intestine of colostrum deprived pigs inoculated with strains of *E. coli* bearing F4, F5, F6, i F41 fimbriae. Arch. Med. Vet. 31, 69–79. doi: 10.4067/S0301-732X1999000100007

[B8] Castro J. Barros M. M. Araújo D. Campos A. M. Oliveira R. Silva S (2022). Swine enteric colibacillosis: Current treatment avenues and future directions. Front. Vet. Sci. 9. doi: 10.3389/fvets.2022.981207, PMID: 36387374 PMC9650617

[B9] Cheng D. Sun H. Xu J. Gao S. (2006). PCR detection of virulence factor genes in *Escherichia coli* isolates from weaned piglets with edema disease and/or diarrhea in China. Vet. Microbiol. 115, 320–328. doi: 10.1016/j.vetmic.2006.02.013, PMID: 16567064

[B10] Connelly S. A. M. AbuOun M. Duggett N. Kirchner M. Navickaite I. Nunez-Garcia J. . (2025). Diversity of antimicrobial resistance and virulence genes of pathogenic *Escherichia coli* recovered from pigs in England. Front. Microbiol. 16. doi: 10.3389/fmicb.2025.1668327, PMID: 41199956 PMC12586179

[B11] Cvetnić S. (2002). “ Kolibaciloza mladunčadi,” in Bakterijske i gljivične bolesti životinja ( Medicinska naklada, Zagreb), 110–124.

[B12] Debroy C. Roberts E. Scheuchenzuber W. Kariyawasam S. Jayarao B. M. (2009). Comparison of genotypes of *Escherichia coli* strains carrying F18ab and F18ac fimbriae from pigs. J. Vet. Diagn. Invest. 21, 359–364. doi: 10.1177/104063870902100310, PMID: 19407090

[B13] Del Carpio A. M. G. Freire C. A. Gomes T. A. T. Abe C. M. Elias W. P. (2025). Importance of aggR sequence variants detection for accurate molecular diagnosis of enteroaggregative *Escherichia coli*. Microbiol. Spectr. 13, e01441–e01425. doi: 10.1128/spectrum.01441-25, PMID: 40990484 PMC12584630

[B14] De Lorenzo C. de Andrade C. P. MaChado V. S. L. Bianchi M. V. Rolim V. M. Cruz R. A. S. . (2018). Piglet colibacillosis diagnosis based on multiplex polymerase chain reaction and immunohistochemistry of paraffin-embedded tissues. J. veterinary Sci. 19, 27–33. doi: 10.4142/jvs.2018.19.1.27, PMID: 28693311 PMC5799396

[B15] de Souza G. F. R. Bueno G. B. de Lira D. R. P. Fernandes I. D. A. Orsi H. Souza B. D. V. L. . (2025). Adherence properties and adhesin-encoding genes detected in enteroaggregative *Escherichia coli (EAEC)*. Microbiol. Spectr. 13 (12), e02070-25. doi: 10.1128/spectrum.02070-25, PMID: 41143428 PMC12671079

[B16] Desvaux M. Dalmasso G. Beyrouthy R. Barnich N. Delmas J. Bonnet R. (2020). Pathogenicity factors of genomic islands in intestinal and extraintestinal *Escherichia coli*. Front. Microbiol. 11. doi: 10.3389/fmicb.2020.02065, PMID: 33101219 PMC7545054

[B17] Do T. N. Wilkie I. Driesen S. J. Fahy V. A. Trott D. J. (2006). Pathogenicity of Vietnamese enterotoxigenic *Escherichia coli* strains in colostrum-deprived one day old piglets. Vet. Pathol. 43, 150–160. doi: 10.1354/vp.43-2-150, PMID: 16537932

[B18] Dubreuil J. D. (2008). *Escherichia coli* STb toxin and colibacillosis: knowing is half the battle. FEMS Microbiol. Lett. 278, 137–145. doi: 10.1111/j.1574-6968.2007.00967.x, PMID: 17995951

[B19] Dubreuil J. D. (2021). Pig vaccination strategies based on enterotoxigenic *Escherichia coli* toxins. Braz. J. Microbiol. 52, 2499–2509. doi: 10.1007/s42770-021-00567-3, PMID: 34244980 PMC8270777

[B20] Dubreuil J. D. Isaacson R. E. Schifferli D. M. (2016). Animal enterotoxigenic *Escherichia coli*. EcoSal Plus 7 (1), 1-47. doi: 10.1128/ecosalplus.ESP-0006-2016, PMID: 27735786 PMC5123703

[B21] EU Reference Laboratory for E. coli (2013). Detection of Enterotoxigenic *Escherichia coli* in food by Real Time PCR amplification of the lt, sth, and stp genes, encoding the heat-labile and heat-stable enterotoxins. Available online at: https://www.iss.it/documents/20126/0/EURL-VTEC_Method_08_Rev+0.pdf/ (Accessed July 15, 2024).

[B22] EU Reference Laboratory for E. coli (2021). Identification of the subtypes of Verocytotoxin encoding genes (vtx) of *Escherichia coli* by conventional PCR. Available online at: https://www.iss.it/documents/20126/0/EURL-VTEC_Method_06_Rev+2.pdf (Accessed July 15, 2024). EU-RL VTEC Method 006 Rev.2.

[B23] Fairbrother J. M. Gyles C. L. (2006). “ *Escherichia coli* infections,” in Diseases of swine, 9th ed. Eds. Straw B. E. Zimmerman J. J. D’Allarie S. Tailor D. J. ( Blackwell Publishing, Ames, Iowa).

[B24] Fairbrother J. M. Nadeau E. (2019). “ Colibacillosis,” in Diseases of swine, 11th. Eds. Zimmerman J. J. Burrough E. R. Karriker L. A. Schwartz K. J. Zhang J. ( John Wiley and Sons, New York).

[B25] Freire C. A. Rodrigues B. O. Elias W. P. Abe C. M. (2022). Adhesin related genes as potential markers for the enteroaggregative *Escherichia coli* category. Front. Cell Infect. Microbiol. 12. doi: 10.3389/fcimb.2022.997208, PMID: 36425788 PMC9679366

[B26] Gibson-Corley K. N. Olivier A. K. Meyerholz D. K. (2013). Priniples for valid histopahtologic scoring in research. Vet. Pathol. 50, 1007–1015. doi: 10.1177/0300985813485099, PMID: 23558974 PMC3795863

[B27] Gomes T. A. Elias W. P. Scaletsky I. C. Guth B. E. Rodrigues J. F. Piazza R. M. . (2016). Diarrheagenic *Escherichia coli*. Braz. J. Microbiol. 47, 3–30. doi: 10.1016/j.bjm.2016.10.015, PMID: 27866935 PMC5156508

[B28] Guerrieri C. G. Pereira M. F. Galdino A. C. M. Dos Santos A. L. S. Elias W. P. Schuenck R. P. . (2019). Typical and Atypical Enteroaggregative *Escherichia coli* Are Both Virulent in the *Galleria mellonella* Model. Front. Microbiol. 10. doi: 10.3389/fmicb.2019.01791, PMID: 31456762 PMC6700222

[B29] Guo L. Wang J. Wang S. Su J. Wang X. Zhu Y. (2020). Characterization of mcr-1–Positive *Escherichia coli* Isolated From Pigs With Postweaning Diarrhea in China. Front. Vet. Sci. 7. doi: 10.3389/fvets.2020.00503, PMID: 33005637 PMC7479848

[B30] Harrington S. M. Dudley E. G. Nataro J. P. (2006). Pathogenesis of enteroaggregative *Escherichia coli* infection. FEMS Microbiol. Lett. 254, 12–18. doi: 10.1111/j.1574-6968.2005.00005.x, PMID: 16451173

[B31] Ikwap K. Larsson J. Jacobson M. Owiny D. O. Nasinyama G. W. NabuKenya I. . (2016). Prevalence of adhesin and toxin genes in *E. coli* strains isolated from diarrheic and non-diarrheic pigs from smallholder herds in northern and eastern Uganda. BMC Microbiol. 16, 178. doi: 10.1186/s12866-016-0796-2, PMID: 27496201 PMC4974785

[B32] Jacobson M. (2022). On the infectious causes of neonatal piglet diarrhoea—A review. Vet. Sci. 9, 422. doi: 10.3390/vetsci9080422, PMID: 36006337 PMC9414921

[B33] Janke B. H. Francis D. H. Collins J. E. Libal M. C. Zeman D. H. Johnson D. D. (1989). Attaching and effacing *Escherichia coli* infections in calves, pigs, lambs and dogs. J. Vet. Diagn. Invest. 1, 6–11. doi: 10.1177/104063878900100104, PMID: 2488649

[B34] Kelly B. G. Vespermann A. Bolton D. J. (2009). Horizontal gene transfer of virulence determinants in selected bacterial foodborne pathogens. Food Chem. Toxicol. 47, 969–977. doi: 10.1016/j.fct.2008.02.007, PMID: 18420327

[B35] Kim K. Song M. Liu Y. Ji P. (2022). Enterotoxigenic *Escherichia coli* infection of weaned pigs: Intestinal challenges and nutritional intervention to enhance disease resistance. Front. Immunol. 13. doi: 10.3389/fimmu.2022.885253, PMID: 35990617 PMC9389069

[B36] Kongsted H. Pedersen K. Hjulsager C. K. Larsen L. E. Pedersen K. S. Jorsa S. E. . (2018). Diarrhoea in neonatal piglets: a case control study on microbiological findings. Porcine Health Manage. 4, 17. doi: 10.1186/s40813-018-0094-5, PMID: 30186621 PMC6120089

[B37] Lerminiaux N. A. Cameron A. D. (2019). Horizontal transfer of antibiotic resistance genes in clinical environments. Can. J. Microbiol. 65, 34–44. doi: 10.1139/cjm-2018-0275, PMID: 30248271

[B38] Li Q. Dai J.-J. Chen S.-Y. Sun R.-Y. Wang D. Bai S.-C. . (2024). Prevalence and molecular characteristics of intestinal pathogenic *Escherichia coli* isolated from diarrheal pigs in Southern China. Vet. Microbiol. 296, 110171. doi: 10.1016/j.vetmic.2024.110171, PMID: 38981202

[B39] Li Y. Qiu X. Li H. Zhang Q. (2007). Adhesive patterns of *Escherichia coli* F4 in piglets of three breeds. J. Genet. Genom. 34, (7) 591–599. doi: 10.1016/S1673-8527(07)60067-8, PMID: 17643944

[B40] Li S. Wang L. Zhou Y. Miao Z. (2020). Prevalence and characterization of virulence genes in *Escherichia coli* isolated from piglets suffering post-weaning diarrhoea in Shandong Province, China. Vet. Med. Sci. 6, 69–75. doi: 10.1002/vms3.207, PMID: 31657876 PMC7036318

[B41] Lin C. S. Huang C. H. Adi V. S. K. Huang C. W. Cheng Y. I. Chen J. H. . (2022). A statistical approach to identify prevalent virulence factors responsible for post-weaning diarrhoeic piglets. Veterinarni Med. 67, 430–439. doi: 10.17221/84/2021-VETMED, PMID: 38846158 PMC11154881

[B42] Luppi A. (2017). Swine enteric colibacillosis: diagnosis, therapy and antimicrobial resistance. Porc Health Manag 3, 16. doi: 10.1186/s40813-017-0063-4, PMID: 28794894 PMC5547460

[B43] Morin N. Santiago A. E. Ernst R. K. Guillot S. J. Nataro J. P. (2013). Characterization of the AggR regulon in enteroaggregative *Escherichia coli*. Infect. Immun. 81, 122–132. doi: 10.1128/IAI.00676-12, PMID: 23090962 PMC3536136

[B44] Nagy B. Arp L. H. Moon H. W. Casey T. A. (1992). Colonization of the small intestine of weaned pigs by enterotoxigenic *Escherichia coli* that lack known colonization factors. Vet. Pathol. 29, 239–246. doi: 10.1177/030098589202900308, PMID: 1621335

[B45] Nagy B. Fekete P. Z. (2005). Enterotoxigenic *Escherichia coli* in veterinary medicine. IJMM. 295, 443–454. doi: 10.1016/j.ijmm.2005.07.003, PMID: 16238018

[B46] Naglić T. Hajsig D. Madić J. Pinter LJ. (2005). Rod *Escherichia*. In Veterinarska mikrobiologija – Specijalna bakteriologija i mikologija. Veterinarski fakultet Zagreb i Hrvatsko mikrobiološko društvo, Zagreb, 58–66.

[B47] Naidoo N. Zishiri O. T. (2025). Presence, pathogenicity, antibiotic resistance, and virulence factors of *Escherichia coli*: A review. Bacteria 4, 16. doi: 10.3390/bacteria4010016

[B48] Nammuang D. Shen Y. W. Ke C. H. Kuan N. L. Lin C. N. Yeh K. S. . (2024). Isolation and evaluation of the pathogenicity of a hybrid shiga toxin-producing and Enterotoxigenic *Escherichia coli* in pigs. BMC veterinary Res. 20, 480. doi: 10.1186/s12917-024-04317-z, PMID: 39434059 PMC11492512

[B49] Nataro J. P. (2005). Enteroaggregative *Escherichia coli* pathogenesis. Curr. Opin. Gastroen. 21, 4–8. 15687877

[B50] Nataro J. P. Steiner T. Guerrant R. L. (1998). Enteroaggregative *Escherichia coli*. Emerg. Infect. Diseases. 4, 251–262. doi: 10.3201/eid0402.980212, PMID: 9621195 PMC2640136

[B51] Nemati A. Gigliucci F. Morabito S. Badouei M. A. (2025). Virulence plasmids in edema disease: Insights from whole-genome analysis of porcine O139:H1 Shiga toxin-producing *Escherichia coli* (STEC) strains. Front. Cell. Infect. Microbiol. 15. doi: 10.3389/fcimb.2025.1528408, PMID: 40182763 PMC11965690

[B52] Paiva R. C. Burrough E. R. Macedo N. Silva A. P. S. P. de Lagarde M. Fairbrother J. M. . (2025). Description of a contemporary pathogenic *Escherichia coli* isolated from pigs with post-weaning diarrhea in the United States from 2010 to 2023. Veterinary Res. 56, 130. doi: 10.1186/s13567-025-01568-y, PMID: 40597240 PMC12218006

[B53] Pakbin B. Brück W. M. Rossen J. W. A. (2021). Virulence factors of enteric pathogenic *Escherichia coli*: A review. Int. J. Mol. Sci. 22, 9922. doi: 10.3390/ijms22189922, PMID: 34576083 PMC8468683

[B54] Paton A. W. Paton J. C. (1998). Detection and characterization of Shiga toxigenic *Escherichia coli* by using multiplex PCR assays for stx1, stx2, eaeA, enterohemorrhagic *E. coli* hlyA, rfbO111, and rfbO157. J. Clin. Microbiol. 36, 598–602. doi: 10.1128/jcm.36.2.598-602.1998, PMID: 9466788 PMC104589

[B55] Penadés J. R. Chen J. Quiles-Puchalt N. Carpena N. Novick R. P. (2015). Bacteriophage-mediated spread of bacterial virulence genes. Curr. Opin. Microbiol. 23, 171–178. doi: 10.1016/j.mib.2014.11.019, PMID: 25528295

[B56] Pokharel P. Dhakal S. Dozois C. M. (2023). The Diversity of *Escherichia coli* Pathotypes and Vaccination Strategies against This Versatile Bacterial Pathogen. Microorganisms 11, 344. doi: 10.3390/microorganisms11020344, PMID: 36838308 PMC9965155

[B57] Robins-Browne R. M. Holt K. E. Ingle D. J. Hocking D. M. Yang J. Tauschek M. (2016). Are *Escherichia coli* pathotypes still relevant in the era of whole-genome sequencing? Front. Cell. infection Microbiol. 6. doi: 10.3389/fcimb.2016.00141, PMID: 27917373 PMC5114240

[B58] Schierack P. Steinrück H. Kleta S. Vajhen W. (2006). Virulence factor gene profiles of *Escherichia coli* isolates from clinicaly healthy pigs. Appl. Environ. Microbiol. 72, 6680–6686. doi: 10.1128/AEM.02952-05, PMID: 17021219 PMC1610323

[B59] Sha W. Beshir Ata E. Yan M. Zhang Z. Fan H. (2024). Swine colibacillosis: analysis of the gut bacterial microbiome. Microorganisms 12, 1233. doi: 10.3390/microorganisms12061233, PMID: 38930615 PMC11205844

[B60] Sugiharto S. Hedemann M. S. Jensen B. B. Lauridsen C. (2012). Diarrhea-like condition and intestinal mucosal responses in susceptible homozygous and heterozygous F4R^+^ pigs challenged with enterotoxigenic *Escherichia coli*. J. Anim. Sci. 90, 281–283. doi: 10.2527/jas.53840, PMID: 23365356

[B61] Tamayo-Legorreta E. García-Radilla A. Moreno-Vázquez E. Téllez-Figueroa F. Alpuche-Aranda C. M. (2021). Diarrheagenic *Escherichia coli* pathotypes isolated from a swine farm in a region of Morelos state, Mexico. Salud Pública México 63, 34–41. doi: 10.21149/11268, PMID: 33984213

[B62] Tsekouras N. Meletis E. Kostoulas P. Labronikou G. Athanasakopoulou Z. Christodoulopoulos G. . (2023). Detection of enterotoxigenic *Escherichia coli* and clostridia in the aetiology of neonatal piglet diarrhoea: important factors for their prevention. Life (Basel Switzerland) 13, 1092. doi: 10.3390/life13051092, PMID: 37240738 PMC10223568

[B63] Tsukahara T. Nakanishi N. Nakayama K. Matsubara N. Ushida K. (2005). Experimental infection of enterotoxemic *Escherichia coli* associated with porcine edema disease and its pathologic characteristics in the intestine. J. Vet. Med. Sci. 67, 1167–1171. doi: 10.1292/jvms.67.1167, PMID: 16327230

[B64] Tusiime M. Mwiine F. N. Afayoa M. Arojjo S. Erume J. (2024). Molecular characterization of *Escherichia coli* virulence markers in neonatal and postweaning piglets from major pig-producing districts of Uganda. BMC veterinary Res. 20, 230. doi: 10.1186/s12917-024-04092-x, PMID: 38802876 PMC11129443

[B65] Van Breda L. K. Dhungyel O. P. Ginn A. N. Iredell J. R. Ward M. P. (2017). Pre- and post-weaning scours in southeastern Australia: A survey of 22 commercial pig herds and characterisation of *Escherichia coli* isolates. PloS One 12, e0172528. doi: 10.1371/journal.pone.0172528, PMID: 28273152 PMC5342203

[B66] Veilleux S. Dubreuil J. D. (2006). Presence of *Escherichia coli* carrying the EAST1 toxin gene in farm animals. Vet. Res. 37, 3–13. doi: 10.1051/vetres:2005045, PMID: 16336921

[B67] Vijtiuk N. Ćurić S. Lacković G. Udovičić I. Vrbanac I. Valpotić I. (1995). Histopathological features in the small intestine of pigs infected with F4ac+ non-enterotoxigenic or enterotoxigenic strains of *Escherichia coli*. J. Comp. Pathol. 112, 1–10. doi: 10.1016/S0021-9975(05)80085-4, PMID: 7722005

[B68] Vu-Khac H. Holoda E. Pilipcinec E. Blanco M. Blanco J. E. Dahbi G. . (2007). Serotypes, virulence genes, intimin types and PFGE profiles of *Escherichia coli* isolated from piglets with diarrhoea in Slovakia. Vet. J. 174, 176–187. doi: 10.1016/j.tvjl.2006.05.019, PMID: 16956777

[B69] Weintraub A. (2007). Enteroaggregative *Escherichia coli*: epidemiology, virulence and detection. J. Med. Microbiol. 56, 4–8. doi: 10.1099/jmm.0.46930-0, PMID: 17172509

[B70] Yamamoto T. Nakazawa M. (1997). Detection and Sequences of the Enteroaggregative *Escherichia coli* Heat-Stable Enterotoxin 1 Gene in Enterotoxigenic *E. coli* Strains Isolated from Piglets and Calves with Diarrhea. J. Clin. Microbiol. 35, 223–227. doi: 10.1128/jcm.35.1.223-227.1997, PMID: 8968912 PMC229543

[B71] Yan X. Huang X. Ren J. Zou Z. Yang S. Ouyang J. . (2009). Distribution of *Escherichia coli* F4 adhesion phenotypes in pigs of 15 Chinese and Western breeds and White Duroc x Erhualian intercross. J. Med. Microbiol. 58, 1112–1117. doi: 10.1099/jmm.0.009803-0, PMID: 19574416

[B72] Yang G.-Y. Guo L. Su J.-H. Zhu Y.-H. Jiao L.-G. Wang J.-F. (2019). Frequency of diarrheagenic virulence genes and characteristics in *Escherichia coli* isolates from pigs with diarrhea in China. Microorganisms 7, 308. doi: 10.3390/microorganisms7090308, PMID: 31480723 PMC6780709

[B73] Zajacova Z. S Faldyna M. Kulich P. Kummer V. Maskova J. Alexa P. (2013). Experimental infection of gnotobiotic piglets with *Escherichia coli* strains positive for EAST1 and AIDA. Vet. Immunol. Immunopathol. 152, 176–182. doi: 10.1016/j.vetimm.2012.09.011, PMID: 23068274

[B74] Zajacova Z. S. Konstantinova L. Aleksa P. (2012). Detection of virulence factors of *Escherichia coli* focused on prevalence of EAST1 toksin in stool of diarrheic and non-diarrheic piglets and presence of adhesion involving virulence factors in *astA* positive strains. Vet. Microbiol. 154, 369–375. doi: 10.1016/j.vetmic.2011.07.029, PMID: 21864997

[B75] Zhang W. Zhao M. Ruesch L. Omot A. Francis D. (2007). Prevalence of virulence genes in *Escherichia coli* strains recently isolated from young pigs with diarrhea in US. Vet. Microbiol. 123, 145–152. doi: 10.1016/j.vetmic.2007.02.018, PMID: 17368762

[B76] Zhu J. Liu Z. Wang S. Gao T. Liu W. Yang K. . (2025). Prevalence, molecular characterization, and antimicrobial resistance profile of enterotoxigenic *Escherichia coli* isolates from pig farms in China. Foods 14, 1188. doi: 10.3390/foods14071188, PMID: 40238372 PMC11989071

[B77] Zweifel C. Schumacher S. Beutin L. Blanco J. Stephan R. (2006). Virulence profiles of Shiga toxin 2e-producing *Escherichia coli* isolated from healthy pigs at slaughter. Vet. Microbiol. 117, 328–332. doi: 10.1016/j.vetmic.2006.06.017, PMID: 16872761

