# Supplementary material for: Impact of virulence genes and pathotypes of intestinal pathogenic Escherichia coli on gastrointestinal lesions in pre- and post-weaning piglets
Source: Front Cell Infect Microbiol. 2026 Jan 26;15:1704407. doi: 10.3389/fcimb.2025.1704407 (PMC12883757; doi:10.3389/fcimb.2025.1704407)
Supplement: Supplementary file 1 [file Table1.docx]

**Supplementary** **Table 1. Isolated bacterial cultures – intestines**. P01-P55 = piglets; SMI = small intestines; LAI = large intestines; Hem = hemolytic *Escherichia coli*; nH = non-hemolytic *Escherichia coli*; Sc ßH = beta-hemolytic *Streptococcus* spp*.*; nc = not cultivated; empty cell = no growth

| PIGLET | SMI  *E.coli* | SMI  other | LAI  *E.coli* | LAI  other | PIGLET | SMI  *E.coli* | SMI  other | LAI  *E.coli* | LAI  other |
| --- | --- | --- | --- | --- | --- | --- | --- | --- | --- |
| P01 | Hem |  | nc | nc | P29 | Hem | *Proteus* | Hem | *Proteus* |
| P02 | Hem |  | nc | nc | P30 | nH |  | nc | nc |
| P03 | nH |  | nc | nc | P31 | Hem |  | Hem |  |
| P04 | nH |  | nc | nc | P32 | Hem |  | Hem |  |
| P05 | Hem |  | nc | nc | P33 | Hem |  | Hem |  |
| P06 | nH |  | nc | nc | P34 | Hem |  | nc | nc |
| P07 | nH |  | nH |  | P35 | Hem |  | Hem |  |
| P08 | nH |  | nH |  | P36 | Hem |  | Hem |  |
| P09 | Hem | *Proteus* | nc | nc | P37 | Hem, nH |  | Hem, nH |  |
| P10 | Hem |  | nc | nc | P38 | nH |  | nH |  |
| P11 | nH |  | nc | nc | P39 | Hem |  | Hem |  |
| P12 | nH |  | nc | nc | P40 | nH |  | nH |  |
| P13 | nH |  | nc | nc | P41 | Hem |  | Hem |  |
| P14 | Hem | *Proteus* | nc | nc | P42 | Hem |  | Hem |  |
| P15 | nH |  | nc | nc | P43 | Hem |  | Hem |  |
| P16 | Hem |  | Hem |  | P44 | Hem | *Proteus* | Hem | *Proteus* |
| P17 | nH |  | nc | nc | P45 | Hem, nH |  | Hem, nH |  |
| P18 | Hem |  | Hem |  | P46 | Hem |  | Hem |  |
| P19 | Hem |  | nc | nc | P47 | Hem, nH |  | Hem, nH |  |
| P20 | nH |  | nc | nc | P48 | nH |  | nH | *Proteus* |
| P21 | Hem |  | nc | nc | P49 | nH |  | nH |  |
| P22 | nH |  | nc | nc | P50 | nH |  | nH |  |
| P23 |  | Sc Hem |  |  | P51 | Hem |  | Hem |  |
| P24 | Hem |  | Hem |  | P52 | Hem |  | Hem |  |
| P25 | Hem |  | Hem |  | P53 | Hem |  | Hem |  |
| P26 | Hem |  | Hem |  | P54 | Hem |  | Hem |  |
| P27 | Hem, nH |  | Hem, nH |  | P55 | nH |  | nH |  |
| P28 | Hem |  | Hem |  |  |  |  |  |  |

**Supplementary Table 2. Molecular characterization of *Escherichia coli* strains***.* 1-58 *E. coli* strains; P01-P55 = piglets; + = virulence gene found; empty cell = virulence gene not found; F4, F5, F6, F18, F41 = fimbrial adhesins; LT = termolabile enterotoxin; STa, STb = thermostable enterorotoxins; Stx1, Stx2, Stx2e = Shiga-toxins; EAST1 = enteroaggregative thermostable enterotoxin; PAA = porcine attaching and effacing associated protein; AIDA-I = adhezin involved in diffuse adherence; ETEC = enterotoxigenic *E. coli*; EPEC = enteropathogenic *E. coli*; STEC = shiga-toxigenic *E. coli*; NSV = non-specific virotype.

|  | | **ADHESINS** | | | | | | | | **TOXINS** | | | | | | | **PATHOTYPE** |
| --- | --- | --- | --- | --- | --- | --- | --- | --- | --- | --- | --- | --- | --- | --- | --- | --- | --- |
|  |  | **fimbrial** | | | | | **non-fimbrial** | | | **termolabile** | | | | **termostable** | | |  |
| **Strain** | **Piglet** | **F4 *faeG*** | **F5 *fanC*** | **F6 *fasA*** | **F18 *fedA*** | **F41 *f41*** | **AIDA-I *aidA*** | **intimin *eae*** | **PAA *paa*** | **LT *eltA*** | **Stx1 *stx1*** | **Stx2 *stx2*** | **Stx2e *stx2e*** | **STa *estI*** | **STb *estII*** | **EAST1 *astA*** |  |
| 1 | P01 |  |  |  |  |  |  |  |  | + |  |  |  | + | + | + | ETEC |
| 2 | P02 |  |  |  |  |  |  |  |  |  |  |  |  |  |  |  |  |
| 3 | P03 |  |  |  |  |  |  |  |  |  |  |  |  |  |  |  |  |
| 4 | P04 |  |  |  |  | + |  |  |  |  |  |  |  |  |  |  | NSV |
| 5 | P05 |  |  |  |  |  |  |  |  | + |  |  |  |  |  | + | ETEC |
| 6 | P06 |  |  |  |  |  | + | + |  |  |  |  |  |  |  |  | EPEC |
| 7 | P07 |  |  |  |  |  |  |  |  |  |  |  |  |  |  |  |  |
| 8 | P08 |  |  |  |  |  |  |  |  |  |  |  |  |  |  |  |  |
| 9 | P09 |  |  |  |  |  |  |  |  |  |  | + | + | + |  |  | ETEC/STEC |
| 10 | P10 |  |  |  |  |  |  |  |  |  |  |  |  |  |  | + | NSV |
| 11 | P11 |  |  |  |  |  |  |  |  |  |  |  |  |  | + |  | NSV |
| 12 | P12 |  |  |  |  |  |  |  |  |  |  |  |  |  |  |  |  |
| 13 | P13 |  |  |  |  |  |  |  |  |  |  |  |  |  |  |  |  |
| 14 | P14 |  |  |  |  |  |  | + |  |  |  |  |  |  |  | + | EPEC |
| 15 | P15 |  |  |  |  |  |  |  |  |  |  |  |  |  |  | + | NSV |
| 16 | P16 | + |  |  |  |  |  |  |  | + |  |  |  |  | + | + | ETEC |
| 17 | P17 |  |  | + |  |  |  |  |  |  |  |  |  | + |  |  | ETEC |
| 18 | P18 |  |  |  |  |  |  |  |  |  |  |  |  |  |  |  |  |
| 19 | P19 | + |  |  | + |  |  |  |  |  |  |  |  | + |  |  | ETEC |
| 20 | P20 |  |  |  |  |  |  |  |  |  |  |  |  |  |  |  |  |
| 21 | P21 | + |  |  |  |  |  |  | + | + |  |  |  | + | + | + | ETEC |
| 22 | P22 |  |  |  |  |  |  |  |  |  |  |  |  |  | + |  | NSV |
| 23 | P24 |  |  |  |  |  |  |  | + | + |  |  |  | + | + | + | ETEC |
| 24 | P25 | + |  |  |  |  |  |  | + | + |  |  |  | + | + | + | ETEC |
| 25 | P26 | + |  |  |  |  |  |  | + | + |  |  |  | + | + | + | ETEC |
| 26 | P27 | + |  |  |  |  |  |  | + | + |  |  |  | + | + | + | ETEC |
| 27 |  |  |  |  |  |  |  |  |  |  |  |  |  |  |  | + | NSV |
| 28 | P28 | + |  |  |  |  |  |  | + | + |  |  |  | + | + | + | ETEC |
| 29 | P29 | + |  | + |  |  |  |  | + | + |  |  |  | + | + | + | ETEC |
| 30 | P30 | + |  |  |  |  |  |  |  |  |  |  |  |  | + | + | ETEC |
| 31 | P31 | + |  |  |  |  |  |  | + | + |  |  |  | + | + | + | ETEC |
| 32 | P32 | + |  |  |  |  |  |  | + | + |  |  |  | + | + | + | ETEC |
| 51 | P33 | + |  |  |  |  |  |  | + | + |  |  |  | + | + | + | ETEC |
| 52 | P34 | + |  |  |  |  |  |  |  |  |  |  |  | + | + | + | ETEC |
| 50 | P35 | + |  |  |  |  |  |  | + | + |  |  |  | + | + | + | ETEC |
| 33 | P36 | + |  |  |  |  |  |  | + | + |  |  |  | + | + | + | ETEC |
| 34 | P37 | + |  |  |  |  |  |  |  |  |  |  |  |  | + | + | ETEC |
| 35 |  |  |  |  |  |  |  |  |  | + |  |  |  |  | + | + | ETEC |
| 36 | P38 | + |  | + |  |  |  |  | + |  |  |  |  | + | + | + | ETEC |
| 37 | P39 |  |  |  |  |  |  |  |  |  |  |  |  |  | + |  | NSV |
| 38 | P40 |  |  |  |  |  |  |  | + |  |  |  |  |  |  | + | NSV |
| 54 | P41 | + |  |  |  |  |  |  | + | + |  |  |  | + | + | + | ETEC |
| 53 | P42 | + |  |  |  |  |  |  | + | + |  |  |  | + | + | + | ETEC |
| 39 | P43 | + |  |  |  |  |  |  | + | + |  |  |  | + | + | + | ETEC |
| 40 | P44 | + |  |  |  |  |  |  | + | + |  |  |  | + | + | + | ETEC |
| 41 | P45 |  |  |  |  |  |  |  |  |  |  |  |  |  |  |  |  |
| 42 |  | + |  |  |  |  |  |  | + | + |  |  |  | + | + | + | ETEC |
| 43 | P46 | + |  | + |  |  |  |  |  | + |  |  |  | + | + |  | ETEC |
| 44 | P47 | + |  | + |  |  |  |  | + |  |  |  |  | + | + | + | ETEC |
| 45 |  | + |  | + |  |  |  |  |  | + |  |  |  |  | + | + | ETEC |
| 46 | P48 | + |  |  |  |  | + |  |  |  |  |  |  |  | + | + | ETEC |
| 47 | P49 | + |  |  |  |  | + |  | + | + |  |  |  |  | + | + | ETEC |
| 48 | P50 | + |  | + |  |  |  |  |  |  |  |  |  |  | + | + | ETEC |
| 49 | P51 | + |  |  |  |  |  |  | + | + |  |  |  | + | + | + | ETEC |
| 55 | P52 | + |  |  |  |  |  |  | + | + |  |  |  | + | + | + | ETEC |
| 56 | P53 | + |  |  |  |  |  |  | + | + |  |  |  | + | + | + | ETEC |
| 57 | P54 | + |  |  |  |  |  |  | + | + |  |  |  | + | + | + | ETEC |
| 58 | P55 |  |  |  |  |  |  |  | + |  |  |  |  |  |  |  | NSV |
|  |  | **32** | **0** | **7** | **1** | **1** | **3** | **2** | **26** | **28** | **0** | **1** | **1** | **29** | **37** | **39** |  |

**Supplementary Table 3. List of piglets with relevant data and presumed cause of death after necropsy**. P01-P55 piglets; F female; M male; S suckling; PW post weaning; + present; F1-F6 farms

| **Piglet** | **Sex** | **Age**  **(days)** | **Weaning** | **Diarrhea** | **Farm** | **Presumed cause of death** |
| --- | --- | --- | --- | --- | --- | --- |
| P01 | F | 21 | S | + | F6 | Catarrhal-hemorrhagic enteritis |
| P02 | F | 21 | S |  | F6 | Interstitial pneumonia |
| P03 | M | 10 | S |  | F5 | Septicemia |
| P04 | M | 26 | S | + | F4 | Septicemia |
| P05 | F | 40 | PW | + | F4 | Catarrhal-hemorrhagic enteritis |
| P06 | M | 80 | PW |  | F4 | Volvulus, fibrinous pleuropneumonia |
| P07 | M | 48 | PW |  | F4 | Hemorrhagic-necrotic pneumonia |
| P08 | F | 32 | PW |  | F4 | Liver rupture, hemoabdomen |
| P09 | F | 32 | PW | + | F4 | Mucous enteritis, polyserositis |
| P10 | M | 13 | S | + | F5 | Mucous enteritis |
| P11 | M | 16 | S |  | F5 | Mucous enteritis, unilateral pyelonephritis |
| P12 | F | 23 | S |  | F2 | Septicemia |
| P13 | M | 38 | PW |  | F6 | Fibrinous-purulent bronchopneumonia |
| P14 | F | 45 | PW |  | F3 | Segmental hemorrhagic enteritis, septicemia |
| P15 | M | 60 | PW | + | F6 | Mucous enteritis, liver rupture |
| P16 | M | 38 | S |  | F3 | Catarrhal-hemorrhagic enteritis, volvulus |
| P17 | F | 5 | S |  | F2 | Mucous gastroenteritis |
| P18 | M | 5 | S |  | F2 | Cystic renal dysplasia |
| P19 | M | 37 | PW |  | F4 | Mucous enteritis |
| P20 | F | 60 | PW |  | F4 | Septicemia |
| P21 | M | 43 | PW | + | F2 | Mucous enteritis |
| P22 | F | 7 | S |  | F3 | Mucous enteritis |
| P23 | M | 60 | PW |  | F3 | Fibrinous polyserositis |
| P24 | M | 6 | S |  | F5 | Catarrhal-hemorrhagic enteritis |
| P25 | F | 6 | S |  | F5 | Catarrhal-hemorrhagic enteritis |
| P26 | F | 6 | S |  | F5 | Catarrhal-hemorrhagic enteritis |
| P27 | M | 8 | S |  | F5 | Catarrhal-hemorrhagic enteritis |
| P28 | M | 3 | S | + | F5 | Catarrhal-hemorrhagic enteritis |
| P29 | F | 3 | S | + | F5 | Catarrhal-hemorrhagic enteritis |
| P30 | F | 75 | PW | + | F4 | Mucous enteritis |
| P31 | M | 2 | S | + | F6 | Hemorrhagic enterocolitis |
| P32 | F | 2 | S | + | F6 | Hemorrhagic enterocolitis |
| P33 | F | 2 | S | + | F6 | Catarrhal-hemorrhagic enteritis, septicemia |
| P34 | F | 82 | PW |  | F4 | Volvulus |
| P35 | M | 9 | S | + | F6 | Catarrhal-hemorrhagic enteritis |
| P36 | M | 9 | S | + | F6 | Catarrhal-hemorrhagic enteritis |
| P37 | M | 20 | S |  | F4 | Septicemia |
| P38 | F | 20 | S |  | F4 | Septicemia |
| P39 | F | 77 | PW |  | F4 | Hemorrhagic gastroenteritis. |
| P40 | F | 2 | S | + | F3 | Segmental hemorrhagic enteritis |
| P41 | F | 13 | S | + | F6 | Hemorrhagic enterocolitis |
| P42 | F | 13 | S | + | F6 | Mucous enteritis |
| P43 | M | 13 | S | + | F6 | Mucous gastroenteritis |
| P44 | M | 14 | S |  | F2 | Mucous enteritis, erosive gastritis |
| P45 | F | 14 | S |  | F2 | Mucous enteritis, hemorrhagic gastritis |
| P46 | M | 4 | S |  | F5 | Mucous enteritis |
| P47 | M | 4 | S |  | F5 | Mucous enteritis |
| P48 | F | 2 | S |  | F5 | Catarrhal-hemorrhagic enteritis |
| P49 | M | 20 | S | + | F1 | Catarrhal-hemorrhagic enteritis |
| P50 | M | 20 | S | + | F1 | Catarrhal-hemorrhagic enteritis |
| P51 | F | 55 | PW |  | F5 | Septicemia |
| P52 | M | 4 | S |  | F1 | Mucous enteritis |
| P53 | F | 4 | S |  | F1 | Mucous enteritis |
| P54 | F | 4 | S |  | F1 | Mucous enteritis |
| P55 | M | 35 | PW | + | F2 | Mucous gastroenteritis |

**Supplementary Table 4: Intensity of histological lesions in piglets.** Abbreviations in table: ' **–** ' not examined; SMI = small intestines; LAI = large intestines; MLN = mesentery lymph nodes; STO = stomach; MES = mesentery

| PIGLET (P) | **GRADE OF MICROSCOPIC LESIONS (0-4)** | | | | | AVERAGE  PER PIGLET |
| --- | --- | --- | --- | --- | --- | --- |
| ORGAN | SMI | MLN | LAI | STO | MES |  |
| P01 | 4 | 1 | 4 | 3 | - | 3,00 |
| P04 | 4 | 4 | 0 | - | 1 | 2,25 |
| P05 | 4 | - | - | - | - | 4,00 |
| P06 | 4 | 3 | - | - | - | 3,50 |
| P09 | 4 | 1 | - | - | 4 | 3,00 |
| P10 | 4 | - | 2 | 0 | 2 | 2,67 |
| P11 | 2 | - | 2 | - | 2 | 2,00 |
| P14 | 3 | 3 | 2 | 1 | 1 | 2,00 |
| P15 | 4 | 1 | - | - | 1 | 2,00 |
| P16 | 4 | - | 4 | 4 | 4 | 4,00 |
| P17 | 2 | - | 3 | 0 | - | 1,66 |
| P19 | 3 | 1 | 2 | 4 | 2 | 2,40 |
| P21 | 4 | 3 | 3 | 2 | - | 3,00 |
| P22 | 3 | 3 | 3 | 1 | - | 2,50 |
| P24 | 4 | 3 | 3 | 3 | - | 3,25 |
| P25 | 4 | 3 | - | 3 | 1 | 2,75 |
| P26 | 4 | 3 | 3 | 2 | 1 | 2,60 |
| P27 | 4 | 3 | 3 | 2 | 1 | 2,60 |
| P28 | 4 | 4 | 4 | 3 | - | 3,75 |
| P29 | 4 | 4 | 3 | 4 | 2 | 3,40 |
| P30 | 4 | 4 | 3 | - | - | 3,67 |
| P31 | 4 | 4 | 2 | 4 | - | 3,50 |
| P32 | 4 | 4 | 2 | - | 2 | 3,00 |
| P33 | 4 | 4 | 3 | - | - | 3,67 |
| P34 | 4 | - | - | - | - | 4,00 |
| P35 | 4 | 4 | 2 | 2 | 2 | 2,80 |
| P36 | 4 | 4 | 2 | 2 | 2 | 2,80 |
| P37 | 4 | 4 | 4 | - | 2 | 3,50 |
| P38 | - | 1 | 3 | - | - | 2,00 |
| P39 | 4 | 2 | 4 | 1 | - | 2,75 |
| P40 | 4 | 3 | 3 | 2 | 4 | 3,20 |
| P41 | 4 | 4 | 4 | 4 | 4 | 4,00 |
| P42 | 4 | 3 | 3 | 4 | 4 | 3,60 |
| P43 | 4 | 3 | 4 | 2 | 1 | 2,80 |
| P44 | 4 | 3 | 4 | 4 | 2 | 3,40 |
| P45 | 4 | 4 | 2 | 3 | 2 | 3,00 |
| P46 | 3 | 3 | 3 | 4 | 2 | 3,00 |
| P47 | 3 | 3 | 4 | 4 | 2 | 3,20 |
| P48 | 4 | 3 | 0 | 0 | - | 1,75 |
| P49 | 4 | 3 | 3 | 0 | 2 | 2,40 |
| P50 | 4 | 3 | 3 | 1 | 2 | 2,60 |
| P51 | 4 | 3 | 4 | - | 1 | 3,00 |
| P52 | 4 | 3 | 3 | 1 | 1 | 2,40 |
| P53 | 4 | 4 | 3 | 1 | 2 | 2,80 |
| P54 | 4 | 4 | 4 | 4 | 2 | 3,60 |
| P55 | 4 | 4 | 3 | 2 | 1 | 2,80 |
| AVERAGE  PER ORGAN | **3,80** | **3,10** | **2,90** | **2,33** | **1,94** | **-** |
